# Identification of *Withania somnifera-Silybum marianum-Trigonella foenum-graecum* Formulation as a Nutritional Supplement to Contrast Muscle Atrophy and Sarcopenia

**DOI:** 10.3390/nu13010049

**Published:** 2020-12-26

**Authors:** Laura Salvadori, Manuela Mandrone, Tommaso Manenti, Catia Ercolani, Luca Cornioli, Mariacaterina Lianza, Paola Tomasi, Sara Chiappalupi, Ester Sara Di Filippo, Stefania Fulle, Ferruccio Poli, Guglielmo Sorci, Francesca Riuzzi

**Affiliations:** 1 Department Translational Medicine, University of Piemonte Orientale, 28100 Novara, Italy; laura.salvadori@uniupo.it; 2 Interuniversity Institute of Myology (IIM), 06132 Perugia, Italy; sara.chiappalupi@unipg.it (S.C.); es.difilippo@unich.it (E.S.D.F.); stefania.fulle@unich.it (S.F.); guglielmo.sorci@unipg.it (G.S.); 3 Department Pharmacy and Biotechnology (FaBiT), University of Bologna, 40126 Bologna, Italy; manuela.mandrone2@unibo.it (M.M.); mariacaterina.lianz3@unibo.it (M.L.); paola.tomasi3@unibo.it (P.T.); ferruccio.poli@unibo.it (F.P.); 4 Biokyma srl Laboratories, 52031 Anghiari, Italy; tmanenti@biokyma.com (T.M.); catia.ercolani@biokyma.com (C.E.); lcornioli@biokyma.com (L.C.); 5 Department Medicine and Surgery, University of Perugia, 06132 Perugia, Italy; 6 Department Neuroscience, Imaging and Clinical Sciences, University G. D’Annunzio Chieti e Pescara, 66100 Chieti, Italy

**Keywords:** skeletal muscle atrophy, medical plant extracts, cytokines, malnutrition, glucocorticoids, signaling pathways

## Abstract

**Background:** Muscle atrophy, i.e., the loss of skeletal muscle mass and function, is an unresolved problem associated with aging (sarcopenia) and several pathological conditions. The imbalance between myofibrillary protein breakdown (especially the adult isoforms of myosin heavy chain, MyHC) and synthesis, and the reduction of muscle regenerative potential are main causes of muscle atrophy. **Methods:** Starting from one-hundred dried hydroalcoholic extracts of medical plants, we identified those able to contrast the reduction of C2C12 myotube diameter in well-characterized in vitro models mimicking muscle atrophy associated to inflammatory states, glucocorticoid treatment or nutrient deprivation. Based on their ability to rescue type II MyHC (MyHC-II) expression in atrophying conditions, six extracts with different phytochemical profiles were selected, mixed in groups of three, and tested on atrophic myotubes. The molecular mechanism underpinning the effects of the most efficacious formulation, and its efficacy on myotubes obtained from muscle biopsies of young and sarcopenic subjects were also investigated. **Results:** We identified WST (*Withania somnifera, Silybum marianum, Trigonella foenum-graecum)* formulation as extremely efficacious in protecting C2C12 myotubes against MyHC-II degradation by stimulating Akt (protein kinase B)-dependent protein synthesis and p38 MAPK (p38 mitogen-activated protein kinase)/myogenin-dependent myoblast differentiation. WST sustains trophism in C2C12 and young myotubes, and rescues the size, developmental MyHC expression and myoblast fusion in sarcopenic myotubes. **Conclusion:** WST strongly counteracts muscle atrophy associated to different conditions in vitro. The future validation in vivo of our results might lead to the use of WST as a food supplement to sustain muscle mass in diffuse atrophying conditions, and to reverse the age-related functional decline of human muscles, thus improving people quality of life and reducing social and health-care costs.

## 1. Introduction

Skeletal muscle atrophy is a complex and highly regulated process characterized by a substantial decrease in muscle mass, strength and regenerative capacity, together with increased myocyte apoptosis [1,2]. Muscle atrophy is associated with aging (sarcopenia), disuse, diabetes, denervation, cancer (cachexia), and several diseases characterized by systemic chronic inflammation, in which muscle atrophy contributes to morbidity and mortality. Muscle atrophy also occurs systematically in response to fasting/nutrient deprivation, a common condition in aged people and critically ill patients, and prolonged therapeutic treatment with glucocorticoids (GCs), a widely medication used for a variety of neoplastic and chronic inflammatory diseases [3]. Despite muscle atrophy represents an enormous medical problem that complicates the diseases in which it occurs, increases hospitalization, and worsens quality of patients’ life, an efficacious therapy is still lacking. Regardless of etiology, the increase of a catabolic state resulting in the breakdown of myofibrillary proteins, especially adult MyHC isoforms, by the activation of the UPS (ubiquitin-proteasome system) and ALS (autophagy-lysosome system), as well as the decrease of muscle protein synthesis [1,2] seem to be necessary to induce muscle atrophy. Atrophying stimuli activate several catabolic pathways [i.e., p38 MAPK (p38 mitogen-activated protein kinase), ERKs (extracellular signal-regulated kinases) and JNK (c-Jun N-terminal kinase)] and transcription factors [such as NF-κB (nuclear factor kappa-light-chain-enhancer of activated B cells)] which are involved in the induction of the muscle-specific ubiquitin ligases, atrogin-1 or *Fbxo32* (*muscle atrophy F-box protein*) and MuRF-1 (muscle RING finger-1) or *Trim63* (*Tripartite motif containing 63*), known as atrogenes, leading to muscle proteolysis. At the same time, the main anabolic pathway, PI3K (phosphoinositide 3-kinase)/Akt (protein kinase B)/mTOR (mammalian target of rapamycin) is deactivated by atrophying stimuli [1,2]. Some pathways involved in the maintenance of muscle trophism, including p38 MAPK and Akt have been shown to play a determinant role in myoblast survival and differentiation [4].

Several bioactive compounds of plant origin have shown ability to reduce muscle inflammation, oxidative stress, fatigue, and damage and increasing muscle differentiation and regeneration [5]. However, results in literature are limited to a few types of medical plants, and their molecular mechanisms of action have not been fully investigated.

We tested one hundred dried hydroalcoholic extracts from medical plants in C2C12 myotubes in order to select plants without toxic effect in normal condition and subsequently, we evaluated their activity in well-characterized in vitro experimental models mimicking muscle atrophy, i.e., treatment of C2C12 myotubes with proinflammatory cytokines TNFα (tumor necrosis factor α)/IFNγ (interferon γ) mimicking muscle atrophy induced by an inflammatory status [6], excess of the glucocorticoid dexamethasone (Dex) or nutrient deprivation (starvation) [7]. We identified a multi-extract formulation strongly efficacious against muscle atrophy associated to different conditions in vitro, and suitable to develop a herbal product to be marketed after further investigation in vivo.

## 2. Materials and Methods

### 2.1. Plant Material

Plant material was provided by Laboratory Biokyma S.r.l, Anghiari (AR), (Italy) from different countries (Table S1) and identified by Dr. Franco Maria Bini. Laboratory Biokyma ensured the quality of the medicinal plant in accordance with UNI EN ISO 9001 quality management certification. The food grade was checked by microbiological and chemical analysis. Each plant was processed in order to decrease the water content down to 10%; to isolate the plant organ containing the highest concentration of active principle (vegetal drug); and to finally obtain a powdered product. Vouchers of crude drugs were deposited in Department of Pharmacy and Biotechnology, University of Bologna (via Irnerio 42, Bologna, Italy) to produce a standardized dry hydroalcoholic extract from each plant as reported in Table S1. The same batch of product were used in the various experiments.

### 2.2. Preparation of the Extracts and the Formulations

Thirty mg of dried and powdered plant material were extracted by sonication for 30 min using 1.5 mL of 50% EtOH. The samples were centrifuged for 20 min and the supernatant was dried to yield the crude extracts, which were firstly solubilized in water at a concentration of 10 mg/mL, and then opportunely diluted to be tested at final concentration of 100 µg/mL in the bioassays. For each sample four extracts were prepared in order to obtain the adequate number of replicates for the bioactivity tests.

In a second step, to test the combined bioactivity of different extracts, the six most efficacious extracts were added to the culture media in groups of three in a 1:1:1 (w/w/w) ratio at a concentration of 33.33 µg/mL for each extract (total concentration of the mix, 100 µg/mL).

### 2.3. Cell Cultures

Murine C2C12 myoblasts were grown in high glucose (4500 mg/L) Dulbecco’s Modified Eagle’s Medium (DMEM, Gibco) supplemented with 20% fetal bovine serum (FBS, Gibco), 100 U/mL penicillin and 100 mg/mL streptomycin (P/S) (growth medium, GM). Differentiation into myotubes was induced by shifting sub-confluent myoblasts to DMEM supplemented with 2% horse serum (HS, Gibco) (differentiation medium, DM) for 4 days [8]. Single plant extracts (100 µg/mL) or different combinations of three extracts (100 µg/mL total concentration) were added to myotubes in absence or presence of 20 ng/mL of recombinant TNFα (Cell Guidance Systems) plus 100 U/mL of IFNγ (Merck-Millipore), 1 µM of Dex (Sigma-Aldrich, St. Louis, MO, USA) or in starvation condition obtained by removing the differentiation medium and incubating myotubes with PBS (phosphate buffered saline), for the indicated time.

### 2.4. Muscle Samples

Vastus lateralis muscle biopsies were obtained from one young (age 30 years) and two aged sarcopenic (age 74.5 ± 2.5 years) subjects undergoing voluntarily needle-biopsy in accordance to the protocol approved by the Ethics Committee for Biomedical Research, University of Chieti (PROT COET 1884/09 recently renewed doc. n.16 of 05/09/2019). The elderly subjects had a diagnosis of sarcopenia according to the criteria of the Centers for Disease Control and Prevention (CDCP). Biopsies were obtained as described [9], and samples were immediately treated to obtain explants placed in culture, as described [10]. The first mononucleated cells migrated out of the explants within 7 to 13 days from the beginning of the culture, independently of the donor age. Isolated activated satellite cells (i.e., myoblasts) were grown in GM containing Ham’s F-10 (Invitrogen) supplemented with 20% FBS (Hyclone), 20 mM l-glutamine, P/S, and 50 μg/mL gentamycin. To induce differentiation, DMEM was supplemented with 2% HS, P/S, 50 μg/mL gentamycin, 10 μg/mL insulin, and 100 μg/mL apo-transferrin (DM) [11].

### 2.5. May-Grünwald/Giemsa Staining

The cells were fixed and processed as previously described [12]. The cells were acquired (Olympus IX51) at 4× magnification and myotube areas were measured in each photo by the use of Image J software (https://imagej.nih.gov/ij/).

### 2.6. Immunofluorescence (IF) for MyHC-II Expression.

Myotubes cultivated on sterile glass coverslips were fixed with 4% paraformaldehyde (PFA), permeabilized using 0.1% Triton X-100 in PBS, blocked with blocking buffer containing 1% glycine (SERVA) and 3% bovine serum albumin (BSA, Sigma-Aldrich) in PBS, and incubated in a humid chamber overnight at 4 °C with mouse anti-MyHC-II primary antibody (eBiosciences) in PBS containing 3% BSA. The next day, coverslips were incubated with anti-mouse Alexa Fluor 488-conjugated antibody (Thermo Fisher Scientific, Waltham, MA, USA) in PBS containing 3% BSA, in a light-tight humid chamber, and counterstained with DAPI (4′,6-diamidino-2-phenylindole) to visualize the nuclei. Coverslips were mounted with fluorescent mounting medium containing 80% glycerol and 20% PBS, and viewed in an epifluorescence microscope (Leica DMRB) equipped with a digital camera.

### 2.7. Morphometric Evaluations

Fusion index (FI), nuclei per myotube (NpM) and myotube diameters were determined on images of MyHC-II staining at 20× magnification using Image J software as previously described [13]. FI was calculated as (number of nuclei in myotubes containing minimum 3 nuclei/total number of nuclei) × 100 in 5 randomly selected fields per well. NpM were counted in 50 randomly chosen myotubes. Average diameters of at least 100 myotubes from 10 randomly chosen fields for each condition were determined. The width of each myotube was measured at 3 different points along the longitudinal axis of the cell.

### 2.8. Western Blotting

Myotube cultures were lysed in protein extraction buffer described in Chiappalupi et al., 2020 [8]. Equal amounts of total protein extract (20 to 30 µg) were resolved by SDS-PAGE (Sodium Dodecyl Sulphate-PolyAcrylamide Gel Electrophoresis) and transferred to nitrocellulose blots (Protran^TM^, 0.45 μm). Following blocking with 5% nonfat dried milk primary and secondary antibodies were applied as indicated in Table S2. The immune reactions were developed by enhanced chemiluminescence. C-DiGit Blot Scanner (LI-COR, USA) was used for blot analysis.

### 2.9. Real-Time PCR

RNA extraction, reverse-transcription and real-time PCR analyses of mRNA contents were performed as previously described [8]. Calculation was performed with the specific software MXPRO-Mx 3000P (Agilent) in comparison with a standard gene (*Gapdh*). The primers used for real-time PCR analysis are reported in Table S3.

### 2.10. Total Phenolic and Flavonoids Content

The assays were performed in Spectrophotometer Jasco V-530 as described by [14].

### 2.11. NMR (Nuclear Magnetic Resonance) Analysis

For NMR analysis extracts were prepared at a concentration of 10 mg/mL in D_2_O containing 0.1 M phosphate buffer and 0.01% of TMSP (trimethylsilylpropanoic acid) standard. ^1^H NMR spectra were recorded at 25 °C on a Varian Inova 600 MHz NMR instrument (600 MHz operating at the ^1^H frequency) as described by Mandrone et al. 2019 [15].

### 2.12. Statistical Analysis

Quantitative data are presented as means ± SD (standard deviation) or SEM (standard error of the mean) of at least three independent experiments. Counts were performed by three independent operators blind to the treatments. Representative experiments and images are shown unless stated otherwise. Statistical analysis was performed using two-tailed, unpaired *t* test. Samples were compared for their phenolic and flavonoid contents by one-way analysis of variance (ANOVA) performed with ‘aov’ function using ‘stats’ package, followed by Tukey’s honestly difference (HSD) post-hoc test presents in ‘stats’ package [15]. p values < 0.05 were considered statistically significant. Correlations between parameters were examined using the Spearman’s rho correlation test. Statistical analyses were performed using R Studio software (version 1.1.463) based on the R software version 4.0.3.

## 3. Results and Discussion

### 3.1. Selection of Plant Extracts with Protective Effect Against TNFα/IFNγ-Induced Myotube Atrophy

One-hundred hydroalcoholic plant extracts, selected based on the reported effects on muscle, traditional use or casually, were added to C2C12 myotubes for 48 h in order to evaluate their effect on myotube area (Figure S1). Four extracts (*B. vulgaris, V. album, C. scolymus* and *A. montana*) were discarded since they showed a dramatic toxic effect. Interestingly, these plants are known for their strong anti-cancer activity by inducing apoptosis [16,17,18,19], and likely they use a common mechanism to overcome the anti-apoptotic resistance typical of cancer cells and myotubes [20]. Among the remaining 96 extracts, *R. rosea*, *I. paraguariensis* and *E. angustifolia* showed the strongest effect in increasing myotube area (Figure S1), suggesting a stimulation of pathways involved in muscle hypertrophy in normal conditions. In accordance, *R. rosea* is traditionally used to alleviate fatigue [21]; *I. paraguariensis* (yerba mate) accelerates muscle strength recovery after exercise and stimulates mitochondriogenesis [22]; and *E. angustifolia* improves the regeneration process in damaged myocardium [23].

The treatment of C2C12 myotubes with TNFα (20 ng/mL)/IFNγ (100 U/mL) (T/I) is known to induce a pronounced reduction of myotube size through selective degradation of sarcomeric MyHC-II, mimicking muscle atrophy induced by an inflammatory status [6]. C2C12 myotubes treated for 48 h with T/I showed ~30% reduction of total area (Figure S2A). Twelve plant extracts (*P. boldus, S. marianum*, *A. archangelica, R. officinale, C. intybus, P. ginseng, T. foenum-graecum, U. dioica, T. platyphyllos, V. vinifera, W. somnifera* and *S. chinensis*) completely abolished the effect of T/I, with *S. marianum* and *W. somnifera* resulting particularly efficacious (~47% increase in myotube area in the presence of cytokines compared to untreated control) (Figure S2A,B). Five plant extracts (*A. membranaceus, H. procumbens, G. biloba, A. repens* and *T. avellanedae)*, in the presence of which T/I were able to reduce myotube area only up to 10% (Figure S2A, dotted line), were included for further investigation.

**Figure 1:**
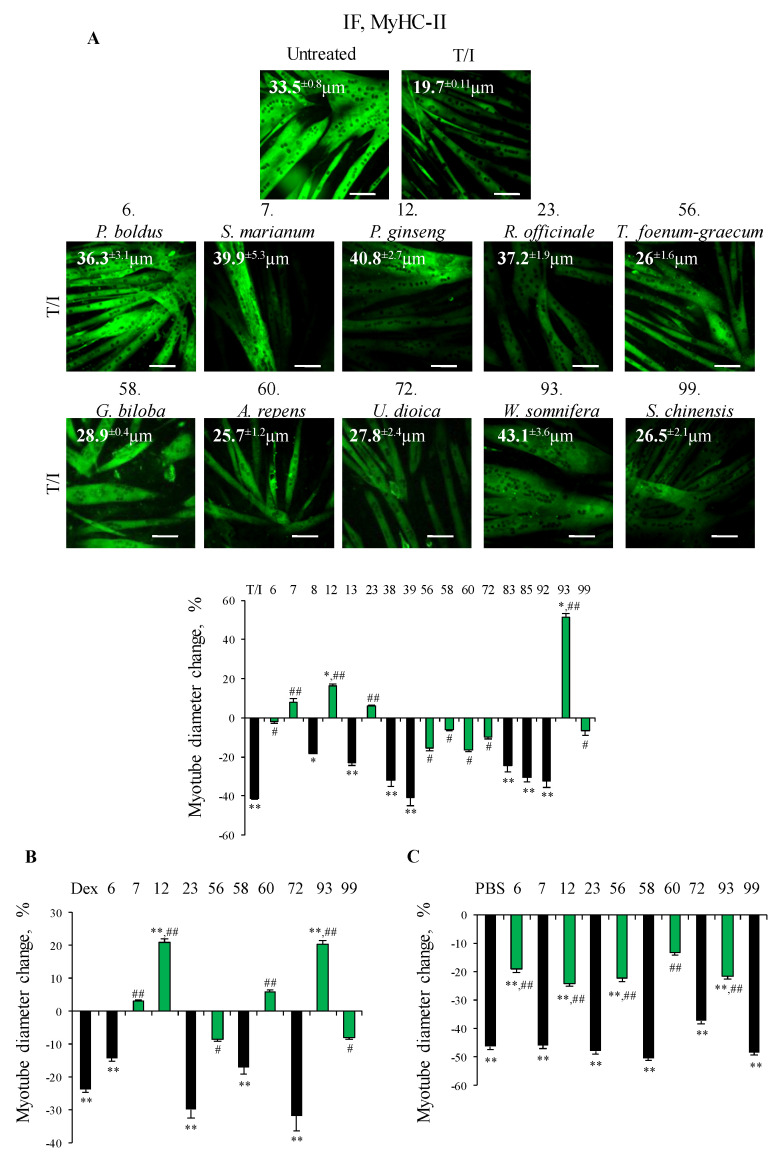
(**A**–**C**) C2C12 myotubes were treated or not with TNFα (tumor necrosis factor α, 20 ng/mL)/IFNγ (interferon γ, 100 U/mL) (T/I), dexamethasone (Dex, 1 µM) or starved with PBS (phosphate buffered saline) in the absence or presence of each selected plant extracts for different times. Immunofluorescence (IF) analysis for myosin heavy chain (MyHC)-II was performed after 48 h or 16 h (PBS), and myotube diameters were measured by Image J software. Reported are representative images with myotube diameters (µm) (**A**) and the percentages of myotube diameters with respect to untreated control (**A**–**C**). The green bars represent the extracts able to protect against myotube atrophy induced by different stimuli. Results are means ± SEM (standard error of the mean) (**A**–**C**). Statistical analysis was conducted using *t*-test * *p* < 0.05, ** *p* < 0.01 significantly different from untreated control; ^#^ *p* < 0.05 and ^##^ *p* < 0.01 significantly different from T/I, Dex or PBS. Bars, 100 µm.

The anti-atrophic effect of the seventeen selected plant extracts was confirmed by measurement of myotube diameters after IF staining for MyHC-II in the presence of T/I. Treatment with T/I reduced myotube diameters by ~40% vs. untreated myotubes (19.7 ± 0.1 vs. 33.5 ± 0.8 µm, respectively) (Figure 1A). *S. marianum, P. ginseng, R. officinale* and *W. somnifera* completely counteracted the effects of T/I, with myotube diameters even increased vs. untreated controls in the presence of *W. somnifera* or *P. ginseng*. Interestingly, silymarin (a mixture of flavonolignans extracted from *S. marianum* seeds) and *W. somnifera* have been proposed as natural intervention for sarcopenia [24,25]. Our data suggest that these plants are able to maintain muscle trophism in the presence of proinflammatory cytokines, which are responsible of the chronic low-grade systemic inflammation, which contributes to the loss of muscle mass in elderly. *P. boldus, T. foenum-graecum, G. biloba*, *A. repens*, *U. dioica* and *S. chinensis* partially counteracted the atrophying stimuli (~2–17% decrease in myotube size in the presence of T/I) (Figure 1A). Of note, *R. officinale*, *A. repens* and *P. boldus* have not been linked to muscle physiology so far, opening to the possibility to use them in muscle wasting conditions.

In conclusion, ten plant extracts appeared able to abolish/reduce the myotube atrophy induced by inflammatory cytokines, and underwent for further investigation.

### 3.2. Selected Plant Extracts Protected Myotubes Against Reduction of Diameter in Different In Vitro Models of Muscle Atrophy

We tested the effects of the ten selected plant extracts in two other well-characterized models of muscle atrophy in vitro consisting of C2C12 myotubes treated with dexamethasone (Dex, 1 µM) for 48 h or nutrient deprivation (PBS) for 16 h [7]. In the presence of Dex, C2C12 myotubes showed a ~24% reduction of their diameter compared to untreated controls (Figure 1B and Figure S3A). As for T/I, the presence of *P. ginseng* and *W. somnifera* resulted in a complete inability of Dex to affect myotube size (average diameters 35.8 ± 2.2 and 35.6 ± 2.5 µm, respectively, i.e., even larger than untreated controls). Treatment with *S. marianum, T. foenum-graecum, A. repens* or *S. chinensis* restrained Dex effect (30.5 ± 2.6, 27.1 ± 1.7, 31.4 ± 2 and 27.2 ± 1.3 µm, respectively), whereas the other extracts resulted inefficacious (Figure 1B and Figure S3A). *P. boldus, P. ginseng, T. foenum-graecum, A. repens* and *W. somnifera* proved protective (only ~20% reduction of myotube diameter compared to control) against the strong (~46%) and rapid reduction of myotube diameter induced by PBS (Figure 1C and Figure S3B).

The maintenance of muscle functionality is linked not only to mass but also to other factors including fibre type composition and the relative isoforms of MyHC. In particular, the myosin fast isoform, MyHC-II, is responsible of skeletal muscle power and speed of movement determining the quality of adult muscles [26]. In the atrophying conditions mimicked by our in vitro experimental models, MyHC-II is the isoform preferentially degraded, and a shift toward the slow isoform, MyHC-I is also reported [27]. The decrease in MyHC-II leads to decrease in shortening velocity and specific tension of the single myofibers translating into reduced skeletal muscle power and speed of movement [26]. Thus, we investigated the effect of the selected extracts against MyHC-II degradation induced by proinflammatory cytokines. Surprisingly, none of the ten selected plants (including *W. somnifera*) was significantly able to preserve MyHC-II expression (Figure S5A), despite a remarkable effect on myotube diameter in the presence of T/I.

### 3.3. ^1^H NMR Profiling and Total Flavonoid Content

**Figure 2:**
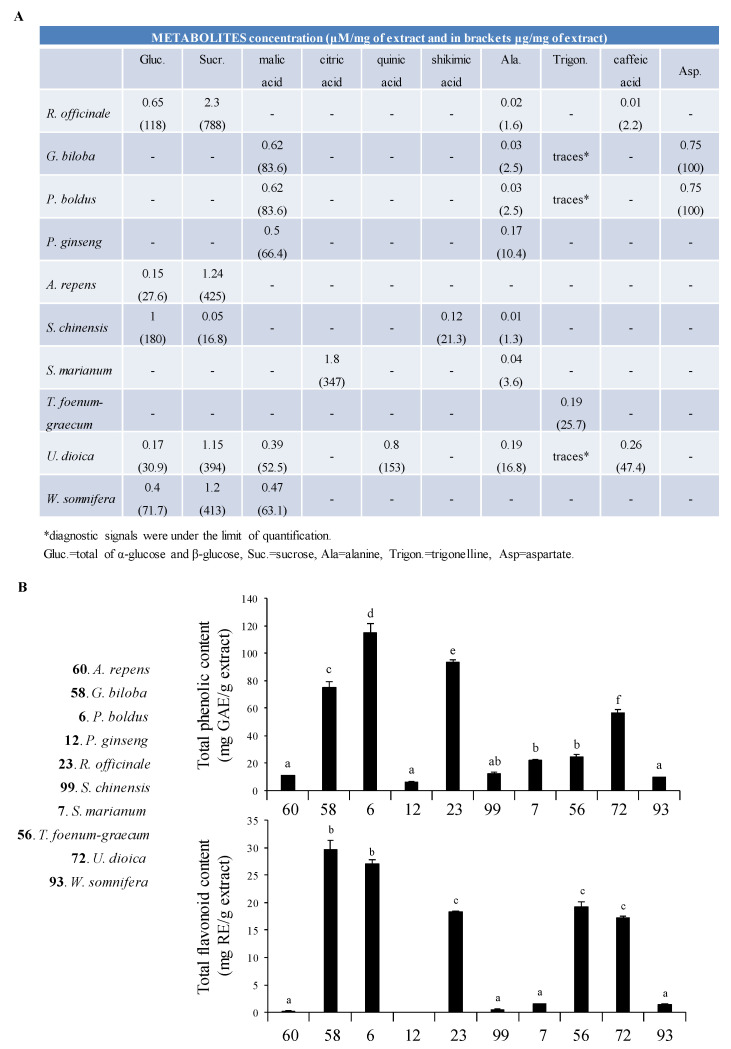
(**A**) Reported are the main metabolites identified in the extracts by ^1^H NMR (nuclear magnetic resonance) profiling and quantified by comparison of metabolites diagnostic signal(s) and TMSP (trimethylsilylpropanoic acid) as internal standards. (**B**) Total phenolic (upper panel) and total flavonoid content (lower panel) of the most active extracts were measured. Different letters within the same assay indicate significant differences by one-way analysis of variance (ANOVA) test (*p* < 0.05). Results are expressed as means ± SD (standard deviation) of three independent experiments.

Since our aim was to propose a herbal product efficient in a wide range of atrophying conditions and able to contrast the degradation of the adult MyHC isoform occurring in muscle atrophy, we decided to analyse the most abundant metabolites contained in the single plant extracts by ^1^H NMR profiling (Figure 2A and Figure S4) in view of obtaining a herbal mixture potentially endowed with synergistic interactions [28]. We found that, except for trigonelline and caffeic acid, the most prominent compounds detected in the extracts were primary metabolites, such as carbohydrates, amino acids, and organic acids. A relevant concentration of caffeic acid was found in *U. dioica* (47.5 μg/mL calculated by semi-quantitative NMR analysis). The same metabolite was present also in *A. repens* at lower level (2 μg/mL) than *U. dioica*. *T. foenum-graecum* was characterized by the presence of the secondary metabolite trigonelline (25.7 μg/mL). The spectrum of *P. ginseng* was characterized by the presence of several aliphatic protons, most likely due to the steroidal compounds (ginsenosides) contained in this plant. *G. biloba* and *P. boldus*, followed by *R. officinale, T. foenum-graecum* and *U. dioica*, yielded the highest content of total flavonoids, generally considered important for the overall bioactivity of a plant extract, especially due to their antioxidant potential [29] (Figure 2B).

*P. ginseng*, *W. somnifera*, *A. repens*, *S. marianum* and *S. chinensis*, which are the most potent extracts counteracting Dex-dependent myotube diameter reduction (Figure 1B and Table S4), were characterized by a low content of total phenolic and flavonoid compounds (Figure 2B). We confirmed by Spearman’s rho statistic test that an inverse and significant correlation exists between phenolic (R2 = 0.841; *p* = 0.002) and flavonoid (R2 = 0.744; *p* = 0.01) compounds and the anti-trophic effects of the extracts, evaluated as percent change of myotube diameter in the presence of Dex compared to untreated myotubes (Figure 1B and Figure S3A). Interestingly, corticosteroid-like actions (i.e., anti-inflammatory and hormonal activities) have been attributed to flavonoids [29], justifying the inability of the extracts containing high amounts of these compounds to contrast Dex effects.

### 3.4. Analysis of Mixed Formulations in Different In Vitro Models of Muscle Atrophy

Although we considered all ten selected extracts as valuable to develop a commercial product, such as a food supplement, able to improve muscle functionality taking advantage by different anti-atrophic mechanisms, we decided to narrow the field to six plants. Thus, we combined in groups of three equal amounts of *P. boldus* (B), *S. marianum* (S), *P. ginseng* (G), *U. dioica* (U), *T. foenum-graecum* (T) and *W. somnifera* (W). The selection here done does not exclude the possibility, in a future work, to explore the activity of the other plants in combination. *U. dioica* was selected for the greatest content of caffeic acid since the chlorogenic acid, an ester of caffeic acid, improves glucose uptake, mitochondrial function, and strength in muscles [30]. These evidences suggest that this class of compounds might contribute to the anti-atrophic effect of *U. dioica.* Moreover, aimed at combining plants with diverse phytochemicals, we selected also *T. foenum-graecum* for the peculiar content of alkaloid trigonelline, which possess numerous biological activities [31]. *T. foenum-graecum* is traditionally used as a tonic, and possess ergogenic and anabolic properties, likely due to protein synthesis stimulation by sapogenin diosgenin, a compound with similar-steroidal activity [32]. *W. somnifera* was chosen since it exerted the highest anti-atrophic activity, and *S. marianum* was also chosen for the relevant content of citric acid, which is recognized and utilized as a dietary supplement to eliminate fatigue after physical exercise [33]. *P. boldus* and *P. ginseng* were considered interesting also for their high content of flavonoids. Moreover, the ginsenoside, Rg1, was shown to prevent muscle protein degradation in starved and Dex-induced atrophic myotubes [34]. Our data confer to *P. ginseng* the ability to contrast cytokine-induced wasting as well.

**Figure 3:**
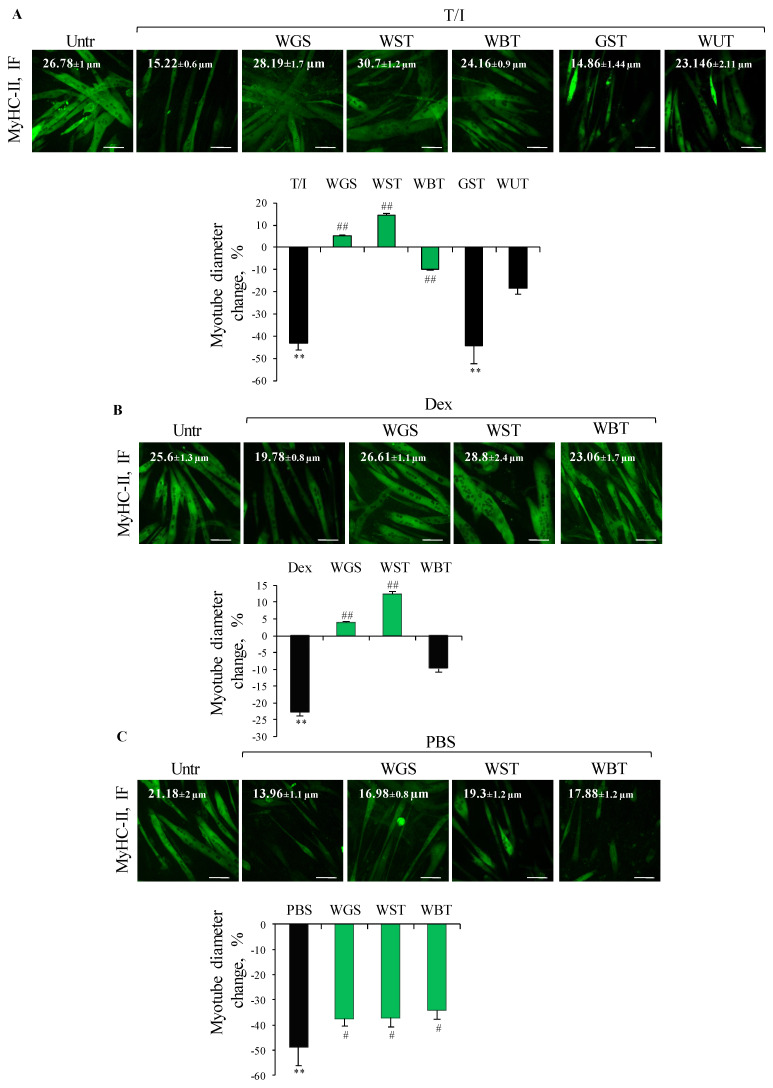
(**A**–**C**) The best formulations were tested (100 µg/mL) on C2C12 myotubes treated or not with TNFα (tumor necrosis factor α, 20 ng/mL)/IFNγ (interferon γ, 100 U/mL) (T/I) or dexamethasone (Dex, 1 µM), or starved with PBS (phosphate buffered saline). After 48 h or 16 h (PBS), immunofluorescence (IF) staining for myosin heavy chain (MyHC)-II was performed and myotube diameters were measured. Reported are representative images with myotube diameters (µm) and the percent changes of myotube diameters with respect to untreated control. The green bars represent the formulations able to protect against myotube atrophy induced by different stimuli. Results are means ± SEM (standard error of the mean) (**A**–**C**). Statistical analysis was conducted using *t*-test ** *p* < 0.01 significantly different from untreated control; ^#^ *p* < 0.05 and ^##^ *p* < 0.01 significantly different from T/I (**A**), Dex (**B**) or PBS (**C**). Bars, 100 µm.

Five of the twenty formulations obtained by mixing the six selected plants (i.e., WGS, WST, WUT, WBT and GST) completely prevented the T/I-induced reduction of myotube area (Figure S5B), but only WGS, WST and WBT were able to counteract the reduction of myotube diameter (Figure 3A). Subsequently WGS, WST and WBT formulations were evaluated in other atrophying conditions (i.e., Dex and starvation) (Figure 3B,C). WST emerged as the most efficacious formulation in preserving myotube diameter in all the atrophying conditions tested (14.6% vs. −43.2%, 12.4% vs. −22.7%, and −37.2% vs. −48.6% change in myotube diameter compared to untreated controls, in the presence of T/I, Dex and starvation, respectively) (Figure 3A–C).

### 3.5. WST Formulation Sustains the Activity of the Anabolic Kinase Akt and Myoblast Differentiation in Different In Vitro Models of Muscle Atrophy

**Figure 4:**
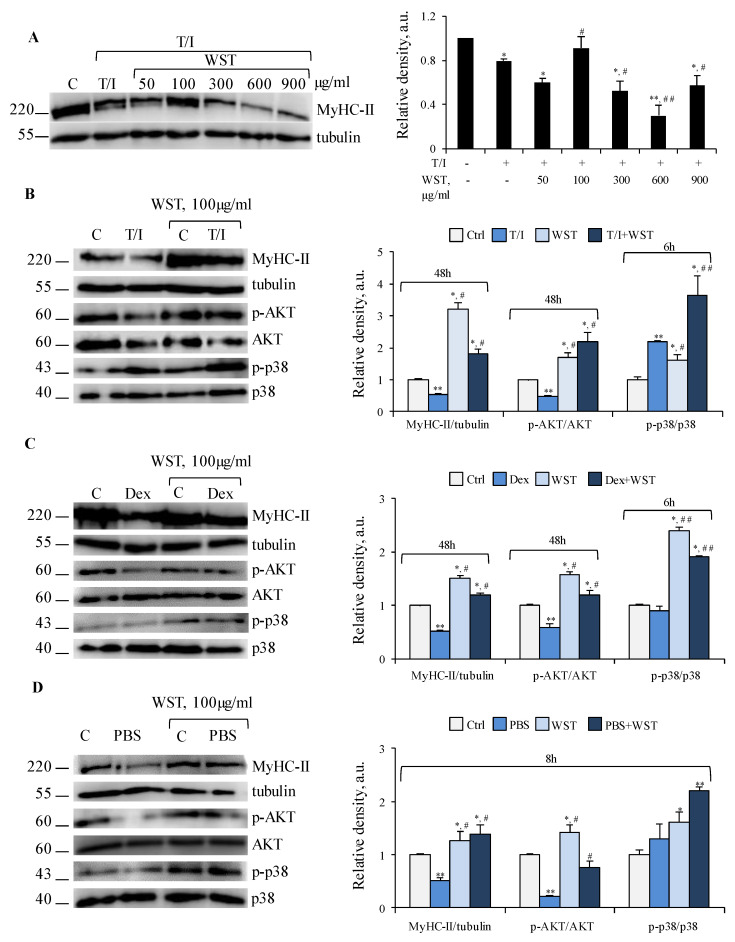
(**A**) C2C12 myotubes were treated with TNFα (tumor necrosis factor α, 20 ng/mL)/IFNγ (interferon γ, 100 U/mL) (T/I) in the absence or presence of different doses of WST for 48 h and myosin heavy chain (MyHC)-II expression were analyzed by WB (western blotting). (**B**–**D**) WST was tested on myotubes untreated or treated with T/I (**B**) or dexamethasone (Dex, 1 µM) (**C**), or starved with PBS (phosphate buffered saline) (**D**) for indicated time-points. MyHC-II, p-AKT (phospho-protein kinase B), AKT, p-p38 MAPK (phosphor-p38 mitogen-activated protein kinase) and p38 MAPK expression were analyzed by WB. Tubulin was used as a loading control (**A**–**D**). Reported are representative images and the relative densities with respect to tubulin or total form of phosphorylated protein (**A**–**D**). Results are means ± SD (standard deviation) (**A**–**D**). Statistical analysis was conducted using *t*-test * *p* < 0.05, ** *p* < 0.01 significantly different from untreated control; ^#^ *p* < 0.05 and ^##^ *p* < 0.01 significantly different from T/I (**A**,**B**), Dex (**C**) or PBS (**D**).

A dose-dependent analysis revealed that WST 100 μg/mL was the most efficacious concentration in counteracting T/I-induced reduction of MyHC-II expression (Figure 4A). On the contrary, WST ≥300 μg/mL exerted the opposite effect (Figure 4A), suggesting that an excess of anti-oxidant metabolites in the formulation can result in detrimental effects by reductive stress [35].

In the absence of atrophying stimuli, WST (100 μg/mL) increased the activation state of Akt, mTOR, ERK1/2 and p38 MAPK, upregulated MyHC-II protein and mRNA, and reduced the activation of NF-κB (p65) without affecting the levels of the differentiation marker MyoD (myoblast determination protein 1), concomitantly with significantly increased myotube diameter (Figure 4B–D and Figure S6A–E). Thus, WST exerts trophic effects *per se* in myotubes. T/I, Dex and starvation are known to cause MyHC-II degradation and reduction of myotube size by different mechanisms [1,6,7,8]. In the presence of T/I, WST counteracted the reduction of MyHC-II levels and deactivation (dephosphorylation) of Akt (Figure 4B), without affecting the reduction of MyoD and *Myh2* levels, and the activation state of mTOR, NF-κB (p65) and ERK1/2 (Figure S6A,B). Thus, WST preserves MyHC-II expression in the presence of atrophying stimuli likely by sustaining an Akt-dependent mTOR-independent protein synthesis. In this condition, Akt is likely to regulate protein synthesis by inhibiting the other downstream target, GSK-3 (glycogen synthase kinase 3) as reported during the regeneration of atrophied skeletal muscles [36].

WST blunted also the atrophying effects of Dex in terms of MyoD and MyHC-II expression, Akt activation (Figure 4C and Figure S6C), and *Myh2* and *Fbxo32* levels (Figure S6D), suggesting the ability of WST to reduce UPS activation. Akt activation and preservation of MyHC-II expression were also observed when WST was added to starved myotubes (Figure 4D). Thus, WST counteracts muscle protein degradation induced by different atrophying stimuli by a common Akt-dependent mechanism, with or without interfering with the activation of UPS system.

In all the atrophying conditions tested, WST caused a strong activation of p38 MAPK (Figure 4B–D), one of the principal pathways inducing muscle catabolism [1] but also crucial for myoblast fusion and differentiation [4]. We evaluated the fusion index (FI) as a marker of myogenic differentiation, and the number of nuclei per myotube (NpM) as an indicator of myotube growth by addition of nuclei derived from non-fused myoblasts.

**Figure 5:**
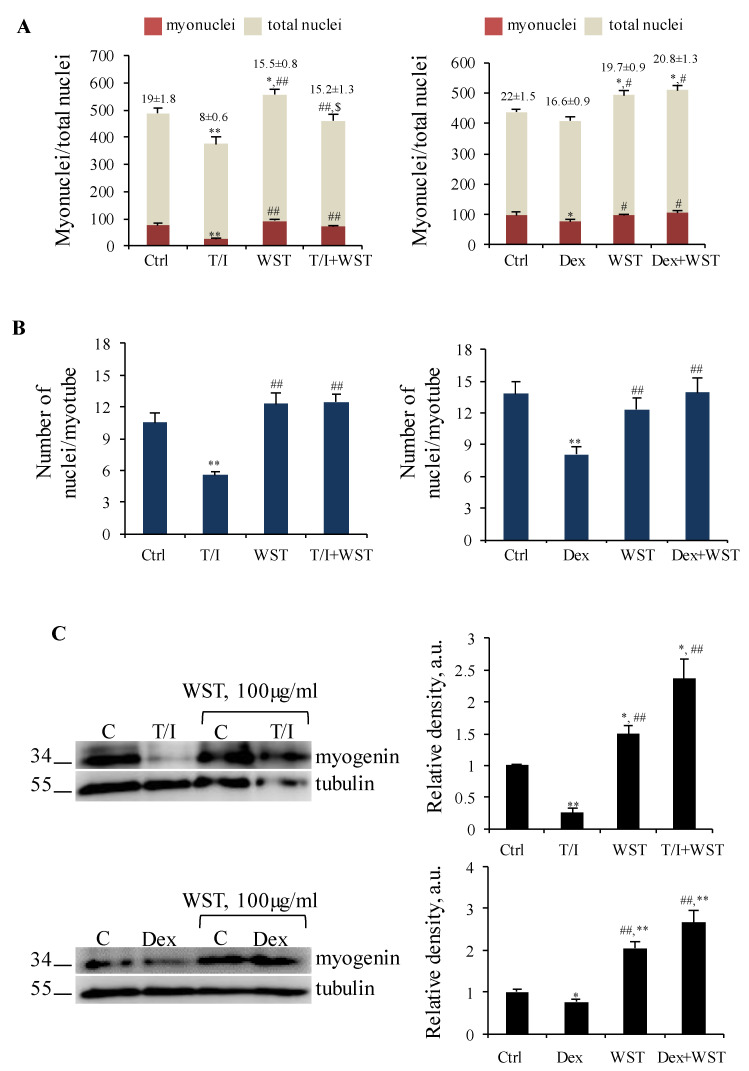
(**A**–**C**) C2C12 myotubes were treated with TNFα (tumor necrosis factor α, 20 ng/mL)/IFNγ (interferon γ, 100 U/mL) (T/I) or dexamethasone (Dex, 1 µM) in the absence or presence of WST for 48 h. The number of nuclei inside myotubes and the total nuclei were counted and the fusion index was calculated and reported (**A**). The average nuclei inside each myotube was calculated (**B**). WB (western blotting) analysis for Myogenin were performed (**C**). Tubulin was used as a loading control (C). Reported are representative images and the relative densities with respect to tubulin (**C**). Results are means ± SD (standard deviation) (**A**–**C**). Statistical analysis was conducted using *t*-test * *p* < 0.05, ** *p* < 0.01 significantly different from untreated control; ^#^ *p* < 0.05 and ^##^ *p* < 0.01 significantly different from T/I or Dex; ^$^ *p* < 0.05 significantly different from WST.

In T/I- or Dex-treated myotubes the FI (8.0% and 16.6%, respectively) and the NpM (5.5 and 8.1, respectively) were significantly lower than in untreated myotubes (~21% FI and ~12 NpM), and WST significantly preserved the myogenic potential and myotube growth (Figure 5A,B).

T/I and Dex also suppressed the expression of the differentiation marker, myogenin (Figure 5C). WST alone increased the expression of myogenin, which was even more up-regulated in the presence of atrophying stimuli, in accordance with the observed high activity of p38 MAPK and expression of MyHC-II compared to control (cfr. Figure 4B,C and Figure 5C).

Collectively, these data suggest that WST formulation is able to contrast muscle atrophy induced by different atrophying stimuli via two common pathways, i.e., p38 MAPK to maintain the myogenic potential and myotube growth, and Akt activation to reduce apoptosis and protein degradation, and sustain muscle trophism preserving myotube diameters and protein content.

### 3.6. WST Formulation Rescues Size and Developmental MyHC (dMyHC) Expression in Human Myotubes Obtained by Sarcopenic Subjects

Sarcopenia is a complex age-related syndrome affecting 40% of adults over 60 and characterized by progressive loss of skeletal muscle mass and strength leading to severe adverse outcomes such as falls, fractures, loss of ambulatory independence, and high hospitalization costs [37]. Malnutrition, treatment with steroid drugs, and low-grade chronic systemic inflammation have been reported as the main causes of muscle protein degradation in sarcopenia. Data obtained in the present work strongly suggest that WST formulation is able to hinder the mechanisms underlying muscle atrophy, also in sarcopenic conditions (Figure 3 and Figure 4), and may represent a useful tool to counteract the age-related decline of muscle tissue.

**Figure 6:**
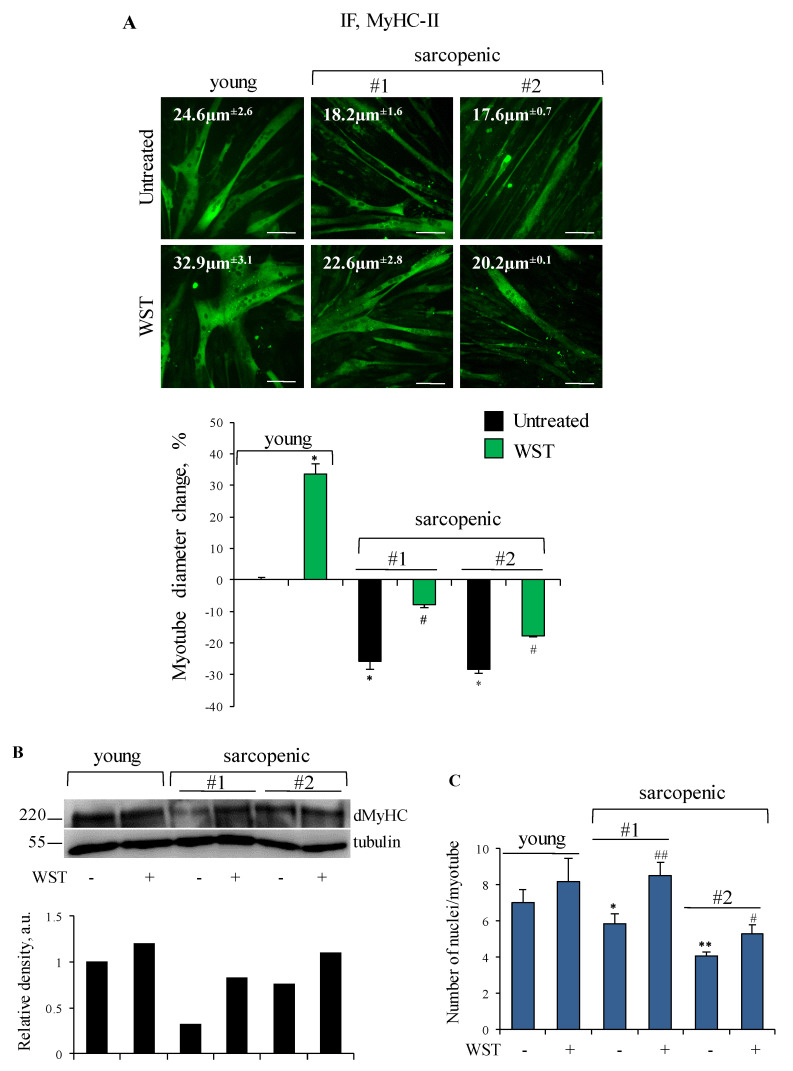
(**A**–**C**) Human myotubes obtained by culturing myoblasts derived from young and sarcopenic subjects (#1 and #2) for 4 days in differentiation medium (DM) were treated or not with WST for 48 h. (**A**) Immunofluorescence (IF) staining for myosin heavy chain (MyHC)-II was performed and myotube diameters were measured. Reported are representative images with indicated the myotube diameters (µm), and the percent changes of myotube diameters with respect to young untreated control. (**B**) Myotubes were lysed to analyze developmental MyHC (dMyHC) expression by WB (western blotting). Reported are representative images and the relative densities with respect to tubulin. (**C**) The average numbers of nuclei/myotube are reported. Results are means ± SEM (standard error of the mean) (**A**,**C**). Statistical analysis was conducted using *t*-test. * *p* < 0.05 and ** *p* < 0.01 significantly different from young untreated control. ^#^ *p* < 0.05 and ^##^ *p* < 0.01 significantly different from internal control. Bars, 100 µm.

Human primary myotubes derived from myoblasts isolated from *Vastus lateralis* muscles of sarcopenic subjects showed smaller size and lower amounts of developmental MyHC (dMyHC) in comparison with those derived from a young subject (Figure 6A,B). Addition of WST to the culture medium resulted in increased diameter of both young and sarcopenic myotubes (Figure 6A), in accordance with the data showed in Figure 4 showing hypertrophic and anti-atrophic effects of the WST formulation. Moreover, WST was able to increase dMyHC expression in sarcopenic myotubes (Figure 6B). In order to establish if the observed increase in myotube size and MyHC expression were the result of hypertrophic effect or increased fusion of myoblasts into myotubes, we evaluated the average number of myonuclei/myotube, and found that in sarcopenic cultures, but not in the young culture, WST formulation stimulates myoblast fusion (Figure 6C). Since the possibility to improve the performance of aged muscle precursor cells by modifying the extracellular environment (i.e., the satellite cell niche) has been reported [38], our result suggests an important rejuvenating effect exerted by the WST formulation, and the possible use of WST as a nutritional supplement to contrast the age-related muscle atrophy, which is largely dependent on the satellite cell efficiency [39].

## 4. Conclusions

Out of one-hundred extracts tested, we identified *P. boldus*, *S. marianum*, *P. ginseng*, *T. foenum-graecum*, *U. dioica* and *W. somnifera* with remarkable ability to prevent/counteract MyHC-II degradation under different atrophying stimuli in vitro. NMR profiling and total phenolic and flavonoid content provided a first overview of the phytochemical composition of the extracts suggesting the presence of multiple metabolites with potential anti-atrophic activity. By mixing the selected extracts in combination of three, we identified WST as a herbal formulation extremely potent in protecting C2C12 myotubes against diameter reduction and MyHC-II degradation under all the atrophying conditions tested by: (i) sustaining MyHC-II synthesis through activation of Akt pathway, independently of mTOR; (ii) activation of p38 MAPK allowing myoblast differentiation; (iii) protecting muscle cells against T/I-induced apoptosis; and (iv) reducing Dex-dependent activation of the UPS.

Malnutrition (fasting or nutrient deprivation), prolonged therapeutic treatments with GCs, or systemic chronic inflammation, are common conditions in aged people predisposing to sarcopenia. Due to the growing life expectancy, sarcopenia represents an urgent and major social and financial problem in Western countries. We showed that WST has a hypertrophic effect on myotubes derived from a young subject and on C2C12 myotubes, with an involvement of the Akt-mTOR pathway in the latter case. Moreover, WST is able to improve the size, dMyHC expression and myoblast fusion in myotubes derived from sarcopenic subjects. Based on our promising in vitro results, further studies in vivo should be performed to evaluate the absorption extent, bioavailability and efficacy of the active compounds contained in the WST formulation. Consequently, a low-cost food supplement could be developed to improve the quality of life of patients and elderly affected by muscle atrophy, also contributing to reduce social and health-care costs.

## Data Availability

The data presented in this study are available on request from the corresponding author. The data are not publicly available in order to preserve the anonymity of the subjects involved in the study.

## Supplementary Materials

The following are available online at https://www.mdpi.com/2072-6643/13/1/49/s1, Table S1: Plants tested in this study, including their vernacular and botanical names, family, organ/s used, and voucher number., Table S2: List of primary and secondary antibodies used in WB, Table S3: List of primers used in real-time PCR.

## References

[1] Bonaldo P., Sandri M. (2013). Cellular and molecular mechanisms of muscle atrophy. Dis. Models Mech..

[2] Scicchitano B.M., Dobrowolny G., Sica G., Musarò A. (2018). Molecular Insights into Muscle Homeostasis, Atrophy and Wasting. Curr. Genom..

[3] Schakman O., Kalista S., Barbé C., Loumaye A., Thissen J.P. (2013). Glucocorticoid-induced skeletal muscle atrophy. Int. J. Biochem. Cell Biol..

[4] Chal J., Pourquié O. (2017). Making muscle: Skeletal myogenesis in vivo and in vitro. Development.

[5] Rondanelli M., Miccono A., Peroni G., Guerriero F., Morazzoni P., Riva A., Guido D., Perna S. (2016). A Systematic Review on the Effects of Botanicals on Skeletal Muscle Health in Order to Prevent Sarcopenia. Evid. Based Complementary Altern. Med..

[6] Ma J.F., Sanchez B.J., Hall D.T., Tremblay A.K., Di Marco S., Gallouzi I.E. (2017). STAT3 promotes IFNγ/TNFα-induced muscle wasting in an NF-κB-dependent and IL-6-independent manner. EMBO Mol. Med..

[7] Sandri M., Sandri C., Gilbert A., Skurk C., Calabria E., Picard A., Walsh K., Schiaffino S., Lecker S.H., Goldberg A.L. (2004). Foxo transcription factors induce the atrophy-related ubiquitin ligase atrogin-1 and cause skeletal muscle atrophy. Cell.

[8] Chiappalupi S., Sorci G., Vukasinovic A., Salvadori L., Sagheddu R., Coletti D., Renga G., Romani L., Donato R., Riuzzi F. (2020). Targeting RAGE prevents muscle wasting and prolongs survival in cancer cachexia. J. Cachexia Sarcopenia Muscle.

[9] Pietrangelo T., D’Amelio L., Doria C., Mancinelli R., Fulle S., Fanò G. (2011). Tiny percutaneous needle biopsy: An efficient method for studying cellular and molecular aspects of skeletal muscle in humans. Int. J. Mol. Med..

[10] Marrone M., La Rovere R.M.L., Guarnieri S., Di Filippo E.S., Monaco G., Pietrangelo T., Bultynck G., Fulle S., Mancinelli R. (2018). Superoxide Anion Production and Bioenergetic Profile in Young and Elderly Human Primary Myoblasts. Oxidative Med. Cell. Longev..

[11] Beccafico S., Riuzzi F., Puglielli C., Mancinelli R., Fulle S., Sorci G., Donato R. (2011). Human muscle satellite cells show age-related differential expression of S100B protein and RAGE. Age.

[12] Sorci G., Riuzzi F., Arcuri C., Giambanco I., Donato R. (2004). Amphoterin stimulates myogenesis and counteracts the antimyogenic factors basic fibroblast growth factor and S100B via RAGE binding. Mol. Cell. Biol..

[13] Baccam A., Benoni-Sviercovich A., Rocchi M., Moresi V., Seelaender M., Li Z., Adamo S., Xue Z., Coletti D. (2019). The Mechanical Stimulation of Myotubes Counteracts the Effects of Tumor-Derived Factors Through the Modulation of the Activin/Follistatin Ratio. Front. Physiol..

[14] Chiocchio I., Mandrone M., Sanna C., Maxia A., Tacchini M., Poli F. (2018). Screening of a hundred plant extracts as tyrosinase and elastase inhibitors, two enzymatic targets of cosmetic interest. Ind. Crop. Prod..

[15] Mandrone M., Antognoni F., Aloisi I., Potente G., Poli F., Cai G., Faleri C., Parrotta L., Del Duca S. (2019). Compatible and incompatible pollen-styles interaction in *Pyrus communis* L. show different transglutaminase features, polyamine pattern and metabolomics profiles. Front. Plant Sci..

[16] Grossarth-Maticek R., Kiene H., Baumgartner S.M., Ziegler R. (2001). Use of Iscador, an extract of European mistletoe (*Viscum album*), in cancer treatment: Prospective nonrandomized and randomized matched-pair studies nested within a cohort study. Altern. Ther. Health Med..

[17] Mileo A.M., Di Venere D., Abbruzzese C., Miccadei S. (2015). Long Term Exposure to Polyphenols of Artichoke (*Cynara scolymus* L.) Exerts Induction of Senescence Driven Growth Arrest in the MDA-MB231 Human Breast Cancer Cell Line. Oxidative Med. Cell. Longev..

[18] Sugier P., Jakubowicz-Gil J., Sugier D., Kowalski R., Gawlik-Dziki U., Kołodziej B., Dziki D. (2020). Chemical Characteristics and Anticancer Activity of Essential Oil from *Arnica montana* L. Rhizomes and Roots. Molecules.

[19] El-Wahab A.E.A., Ghareeb D.A., Sarhan E.E., Abu-Serie M.M., El Demellawy M.A. (2013). In vitro biological assessment of *Berberis vulgaris* and its active constituent, berberine: Antioxidants, anti-acetylcholinesterase, anti-diabetic and anticancer effects. BMC Complementary Altern. Med..

[20] Xiao R., Ferry A.L., Dupont-Versteegden E.E. (2011). Cell death-resistance of differentiated myotubes is associated with enhanced anti-apoptotic mechanisms compared to myoblasts. Apoptosis.

[21] Ishaque S., Shamseer L., Bukutu C., Vohra S. (2012). Rhodiola rosea for physical and mental fatigue: A systematic review. BMC Complementary Altern. Med..

[22] Panza V.P., Diefenthaeler F., Tamborindeguy A.C., Camargo C., de Moura B.M., Brunetta H.S., Sakugawa R.L., de Oliveira M.V., Puel E., Nunes E.A. (2016). Effects of mate tea consumption on muscle strength and oxidative stress markers after eccentric exercise. Br. J. Nutr..

[23] Abdelmonem M., Kassem S.H., Gabr H., Shaheen A.A., Aboushousha T. (2015). Avemar and *Echinacea* extracts enhance mobilization and homing of CD34^+^ stem cells in rats with acute myocardial infarction. Stem Cell Res. Ther..

[24] Raut A.A., Rege N.N., Tadvi F.M., Solanki P.V., Kene K.R., Shirolkar S.G., Pandey S.N., Vaidya R.A., Vaidya A.B. (2012). Exploratory study to evaluate tolerability, safety, and activity of Ashwagandha (*Withania somnifera*) in healthy volunteers. J. Ayurveda Integr. Med..

[25] Kumar J., Park K.C., Awasthi A., Prasad B. (2015). Silymarin extends lifespan and reduces proteotoxicity in C. elegans Alzheimer’s model. CNS Neurol. Disord. Drug Targets.

[26] Larsson L., Li X., Frontera W.R. (1997). Effects of aging on shortening velocity and myosin isoform composition in single human skeletal muscle cells. Am. J. Physiol..

[27] Ciciliot S., Rossi A.C., Dyar K.A., Blaauw B., Schiaffino S. (2013). Muscle type and fiber type specificity in muscle wasting. Int. J. Biochem. Cell Biol..

[28] Panossian A., Hamm R., Kadioglu O., Wikman G., Efferth T. (2013). Synergy and Antagonism of Active Constituents of ADAPT-232 on Transcriptional Level of Metabolic Regulation of Isolated Neuroglial Cells. Front. Neurosci..

[29] Ruskovska T., Maksimova V., Milenkovic D. (2020). Polyphenols in human nutrition: From the in vitro antioxidant capacity to the beneficial effects on cardiometabolic health and related inter-individual variability—An overview and perspective. Br. J. Nutr..

[30] Ong K.W., Hsu A., Tan B.K. (2013). Anti-diabetic and anti-lipidemic effects of chlorogenic acid are mediated by AMPK activation. Biochem. Pharmacol..

[31] Zhou J., Chan L., Zhou S. (2012). Trigonelline: A plant alkaloid with therapeutic potential for diabetes and central nervous system disease. Curr. Med. Chem..

[32] Folwarczn J., Zych M., Nowińska B., Pytli M., Bialik M., Jagusiak A., Lipecka-Karcz M., Matysiak M. (2016). Effect of diosgenin, a steroidal sapogenin, on the rat skeletal system. Acta Biochim. Pol..

[33] Sugino T., Aoyagi S., Shirai T., Kajimoto Y., Kajimoto O. (2007). Effects of Citric Acid and L-Carnitine on Physical Fatigue. J. Clin. Biochem. Nutr..

[34] Li F., Li X., Peng X., Sun L., Jia S., Wang P., Ma S., Zhao H., Yu Q., Huo H. (2017). Ginsenoside Rg1 prevents starvation-induced muscle protein degradation via regulation of AKT/mTOR/FoxO signaling in C2C12 myotubes. Exp. Ther. Med..

[35] Bellezza I., Riuzzi F., Chiappalupi S., Arcuri C., Giambanco I., Sorci G., Donato R. (2020). Reductive stress in striated muscle cells. Cell. Mol. Life Sci..

[36] Pansters N.A., Schols A.M., Verhees K.J., de Theije C.C., Snepvangers F.J., Kelders M.C., Ubags N.D., Haegens A., Langen R.C. (2015). Muscle-specific GSK-3β ablation accelerates regeneration of disuse-atrophied skeletal muscle. Biochim. Biophys. Acta.

[37] Larsson L., Degens H., Li M., Salviati L., Lee Y.I., Thompson W., Kirkland J.L., Sandri M. (2019). Sarcopenia: Aging-Related Loss of Muscle Mass and Function. Physiol. Rev..

[38] Gopinath S.D., Rando T.A. (2008). Stem cell review series: Aging of the skeletal muscle stem cell niche. Aging Cell.

[39] Snijders T., Parise G. (2017). Role of muscle stem cells in sarcopenia. Curr. Opin. Clin. Nutr. Metab. Care.

