# Supplementary material for: Identification of Withania somnifera-Silybum marianum-Trigonella foenum-graecum Formulation as a Nutritional Supplement to Contrast Muscle Atrophy and Sarcopenia
Source: Nutrients. 2020 Dec 26;13(1):49. doi: 10.3390/nu13010049 (PMC7824275; doi:10.3390/nu13010049)
Supplement: Supplementary file 1 [file nutrients-13-00049-s001.pdf]

Supplemental Information

Table S1.

N	Vernacular name	Scientific name	Family	Used plant organ	Origin	Voucher number
1	COMMON FUMITORY	*Fumaria officinalis L.*	Papaveraceae	flowered top	Macedonia	BIOF26733T
2	OLIVE	*Olea europaea L.*	Oleacee	leaves	Italy	BIOF42911T
3	BLACKCURRANT	*Ribes nigrum L.*	Grossulariacee	leaves	Italy	BIOF52511T
4	SAGE	*Salvia officinalis L.*	Lamiaceae	leaves	Italy	BIOF56111T
5	ELDERBERRY	*Sambucus nigra L.*	Adoxaceae	flowers	Bulgaria	BIOF56322I
6	BOLDO	*Peumus boldus Molina*	Monimiaceae	leaves	Chile	BIOU08511T
7	CARDUS MARIANUS	*Silybum marianum Gaert.*	Asteraceae	fruits	Austria	BIOU12155I
8	COMMON CHICORY	*Cichorium intybus L.*	Asteraceae	root	Italy	BIOU15344C
9	ROSELLE	*Hibiscus sabdariffa L.*	Malvaceae	flowers	Egypt	BIOU31922T
10	COMMON MALLOW	*Malva sylvestris L.*	Malvaceae	leaves	Italy	BIOU37311T
11	MACA	*Lepidium meyenii L.*	Brassicaceae	powder root	Peru	BIOZ37044P
12	ASIAN GINSENG	*Panax ginseng Meyer*	Araliaceae	root	China	PC29144T
13	GARDEN ANGELICA	*Angelica archangelica L.*	Apiaceae	root	China	PFU03844C
14	BITTER ORANGE	*Citrus aurantium L. var. dulcis L.*	Rutaceae	flowers	Iran	PFU04922I
15	BITTER ORANGE	*Citrus aurantium L. var. dulcis L.*	Rutaceae	zests	Albania	PFU04967T
16	CHAMOMILE	*Chamomilla recutita Rausch.*	Asteraceae o Compositae	flowers	Italy	PFU10522I
17	CHINESE PARSLEY	*Coriandrum sativum L.*	Apiaceae	fruits	Italy	PFU17955I
18	WILLOWHERBS	*Epilobium angustifolium L.*	Onagraceae	summit	Serbia	PFU21933T
19	HYSSOP	*Hyssopus officinalis L.*	Lamiaceae	flowers and leaves	Italy	PFU31322I
20	LAVENDER	*Lavandula angustifolia Miller*	Lamiaceae	flowers	France	PFU32722I
21	COMMON POPPY	*Papaver rhoeas L.*	Papaveraceae	petals	Albania	PFU44708T
22	BROADLEAF PLANTAIN	*Plantago major L.*	Plantaginaceae	leaves	Poland	PFU46711T
23	CHINESE RHUBARB	*Rheum officinale Baill.*	Polygonaceae	rhizomes	China	PFU51799C
24	DOG ROSE	*Rosa canina L.*	Rosaceae	cinorrodes/ seeds	Hungary	PFU53310I

25	RUSTYBACK	*Ceterach officinarum DC.*	Aspleniaceae	aerial parts	Italy	PFU59533T
26	COMMON THYME	*Thymus vulgaris L.*	Lamiaceae	leaves	Turkey	PFU62311T
27	KINNIKINNICK	*Arctostaphylos uva-ursi Spreng.*	Ericaceae	leaves	Russia	PFU63311T
28	BLACKSAMSON ECHINACEA	*Echinacea angustifolia DC.*	Asteraceae	root	Italy	PFZ20544C
29	OAT	*Avena sativa L.*	Poaceae	aerial parts	Hungary	PS06309T
30	CREAT	*Andrographis paniculata Nees*	Acanthaceae	whole plant	India	PS16409T
31	CACAO TREE	*Theobroma cacao*	Malvaceae	beans	Peru	PS18677I
32	WILD YAM	*Dioscorea villosa*	Dioscoreaceae	root		PS20044P
33	COMMON HOP	*Humuluslu pulus L.*	Cannabaceae	flowers	China	PS36722I
34	CHASTE TREE	*Vitex agnus castus L.*	Verbenaceae o Lamiaceae	fruits	Morocco	PU01355I
35	BITTER ALOE	*Aloe ferox Miller*	Xanthorrhoeace ae	juice	Porth Elisabeth	PU02561P
36	PINEAPPLE	*Ananas sativus L.*	Bromeliaceae	stem	China	PU03555T
37	FEMALE GINSENG	*Angelica sinesi Diels*	Apiaceae	root	Poland	PU04044C
38	GRAPPLE PLANT	*Harpagophytum procumbens DC.*	Pedaliaceae	root	Namibia	PU05544C
39	MILKVETCH	*Astragalus membranaceus Fisch.*	Fabaceae/Legu minosae	root	China	PU06244C
40	GREATER BURDOCK	*Arctium lappa L.*	Asteraceae	root	Poland	PU06744C
41	COMMON BARBERRY	*Berberisvulgaris L.*	Berberidaceae	bark	India	PU07188T
42	HAWTHORN	*Crataegus oxyacantha L.*	Rosaceae	flowers and leaves	Poland	PU08112T
43	SHEPHERD'S BURSE	*Capsella bursa-pastorisMedicus*	Brassicaceae	flowered tops	Albania	PU08933T
44	ROBUSTA COFFEA	*Coffea robusta L.*	Rubiaceae	grains	India	PU09603C
45	CEYLON CINNAMON	*Cinnamomum zeylanicum Nees*	Lauraceae	bark	Ceylon	PU11188T
46	FRENCH ARTICHOKE	*Cynarascolymus Hayek*	Asteraceae	leaves	Italy	PU11511T
47	CENTELLA, ASIATIC PENNYWORT	*Hydrocotyle asiatica L.*	Apiaceae	aerial parts	Madagascar	PU14111T
48	CHINCHONA, QUINA QUINA	*Cinchona succirubra Pav.*	Rubiaceae	bark	Indonesia	PU15188C
49	TURMERIC	*Curcuma longa L.*	Zingiberaceae	rhizomes	India	PU19344C
50	PALE PURPLE CONEFLOWER	*Echinacea pallida Britton*	Asteraceae	root	Italy	PU20444C
51	EASTERN PURPLE CONEFLOWER	*Echinacea purpurea Moench.*	Asteraceae	root	Poland	PU20644C

52	DEVIL'S BUSH	*Eleutherococcus senticosus Maxim.*	Araliaceae	root	China	PU21144C
53	ITALIAN STRAWFLOWER	*Helicrysum italicum G. Don*	Asteraceae	flowered tops	Italy	PU21333T
54	HOARY WILLOWHERB	*Epilobium parviflorum Schreb.*	Onagraceae	flowered tops	Albania	PU21833T
55	COMMON HORSETAIL	*Equisetum arvense L.*	Equisetaceae	stems	Poland	PU22001T
56	FENUGREEK	*Trigonella foenum-graecum L.*	Leguminosae	seeds	India	PU25377I
57	FENNEL	*Foeniculum vulgare Miller*	Apiaceae	fruits	India	PU25677I
58	GINKGO	*Ginkgo biloba L.*	Ginkgoaceae	leaves	Italy	PU28911T
59	BERMUDA GRASS	*Cynodon dactylon L.*	Poaceae	rhizomes	China	PU29499C
60	COMMON COUCH	*Agropyrum repens Beauv.*	Poaceae	rhizomes	Serbia	PU29999C
61	GUARANA'	*Paullini asorbilis Mart.*	Sapindaceae	seeds	Brasil	PU30577I
62	FRANKINCENSE	*Boswelia carterii Birdwood*	Burseraceae	grains	Ethiopia	PU30703I
63	PERFORATE ST JOHN'S-WORT	*Hypericum perforatum L.*	Clusiaceae	flowered tops	Serbia	PU31233T
64	LIQUORICE	*Glycyrrhiza glabra L.*	Fabaceae	root	Egypt	PU34344C
65	YERBA MATE	*Ilex paraguariensis St. Hil.*	Aquifoliaceae	flowered tops	Brasil	PU38533T
66	LEMON BALM	*Melissa officinalis L.*	Lamiaceae	leaves	Italy	PU38911T
67	PEPPERMINT	*Mentha piperita L.*	Lamiaceae	leaves	Serbia	PU39511T
68	COMMON BILBERRY	*Vaccinium myrtillus L*	Ericaceae	leaves	Hungary	PU40511T
69	MORINGA	*Moringa oleifera Lamk.*	Moringaceae	leaves	Burkina Faso	PU40711T
70	MUIRAPUAMA	*Ptychopetalum olacoides Bentham*	Olacaceae	wood	Brasil	PU41104T
71	JAVA TEA	*Orthosiphon stamineus Benth.*	Lamiaceae	leaves	Indonesia	PU43711T
72	COMMON NETTLE	*Urtica dioica L.*	Urticaceae	leaves	Italy	PU43911T
73	BLACK PEPPER	*Piper nigrum L.*	Piperaceae	grains	Brasil	PU46303I
74	CAYENNE PEPPER	*Capsicum annuum L.*	Solanaceae	fruits	Uganda	PU46555I
75	SCOTS PINE	*Pinus sylvestris L.*	Pinaceae	gems	Albania	PU47302I
76	POLLEN mix flower				Spain	PU48903I
77	GOLDEN ROOT	*Rhodiola rosea L.*	Crassulaceae	root	China	PU53244T
78	RED RASPBERRY	*Rubus idaeus L.*	Rosaceae	leaves	Albania	PU54711T
79	CATBRIERS	*Smilax medica Cham. et Sch.*	Smilacaceae	root	Mexico	PU55944C

80	MOUNTAIN SAVORY	*Satureja montana L.*	Lamiaceae	leaves	Albania	PU56911I
81	ALEXANDRIAN SENNA	*Cassia angustifolia Vahl.*	Leguminosae	leaves	India	PU58311T
82	SAW PALMETTO	*Serenoa serrulata Hook.*	Arecaceae	fruits	USA	PU58755T
83	PINK LAPACHO	*Tabebuia avellane daelorentz ex griseb*	Bignoniaceae	bark	Paraguay	PU60244C
84	TEA PLANT	*Camellia sinesi Kuntze*	Theaceae	leaves	China	PU61111I
85	LARGELEAF LINDEN	*Tilia platyphyllos Scop.*	Malvaceae	flowers and bracts	Serbia	PU61533T
86	BOGBEAN	*Menyanthes trifoliata L.*	Menyanthaceae	leaves	North Europe	PU62711T
87	RED CLOVER	*Trifolium pratense L.*	Leguminosae	fruits	Albania	PU62822I
88	CAT'S CLAW	*Uncaria tomentosa DC.*	Rubiaceae	bark	Peru	PU63288T
89	VALERIAN	*Valeriana officinalis L.*	Caprifoliaceae	root	Poland	PU63744C
90	GREAT MULLEIN	*Verbascum thapsus L.*	Scrophulariacea e	flowers	Hungary	PU64122I
91	EUROPEAN MISTLETOE	*Viscum album L.*	Santalaceae	leaves and twings	Hungary	PU65711I
92	COMMON GRAPE VINE	*Vitis vinifera L.*	Vitaceae	leaves	Tunisia	PU66711T
93	INDIAN GINSENG	*Withania somnifera Dunal*	Solanaceae	root	India	PU67044C
94	GINGER	*Zingiber officinale Roscoe*	Zingiberaceae	rhizomes	Nigeria	PU67999T
95	PUMPKINS	*Cucurbita pepo L.*	Cucurbitaceae	seeds	China	PU68277I
96	WOLF'S BANE	*Arnica montana L.*	Asteraceae	flowers	Hungary	PZ05222I
97	DAMIANA	*Turnera diffusa Willd*	Passifloraceae	leaves	Mexico	PZ19711T
98	TARRAGON	*Artemisia dracunculus L.*	Asteraceae	leaves	Poland	PZ19911T
99	MAGNOLIA VINE	*Schisandra chinensis Turcz. Baill.*	Schisandraceae	fruits	China	PZ57455I
100	CALIFORNIA POPPY	*Eschscholzia californica Cham.*	Papaveraceae	whole flowered plant	Italy	BIOD3744I

Table S1. Plants tested in this study, including their vernacular and botanical names, family, organ/s used, and voucher number.

Table S2.

PrimaryAntibody	Molecular weight (kDa)	Dilution	Source
Mouse monoclonal anti-MyHC-II (MF20)	220	1:10000	eBiosciences
Mouse monoclonal anti-MyHC developmental (RNMY2/9D2)	220	1:500	Monosan
Mouse monoclonal anti-MyoD (5.8A)	45	1:500	Santa Cruz Biotech
Mouse monoclonal anti-Myogenin (F5D)	34	1:1000	Santa Cruz Biotech
Mouse monoclonal anti-α-Tubulin (DM1A)	55	1:2000	Santa Cruz Biotech
Rabbit monoclonal anti-phospho-Akt (Ser473) (D9E)	60	1:2000	Cell Signaling Tech.
Rabbit monoclonal anti-phospho-NF-kB p65 (Ser536) (93H1)	65	1:2000	Cell Signaling Tech.
Rabbit monoclonal anti-phospho-p38 MAPK (Thr180/Tyr182) (D3F9) XP	43	1:2000	Cell Signaling Tech.
Rabbit polyclonal anti-phospho-p44/42 MAPK (Erk1/2) (Thr202/Tyr204)	42/44	1:2000	Cell Signaling Tech.
Rabbit polyclonal anti-phospho-mTOR (Ser2448)	289	1:1000	Cell Signaling Tech.
Rabbit polyclonal anti-Akt (Thr308) (D25E6)	60	1:2000	Cell Signaling Tech.
Rabbit polyclonal anti-NF-kB (p65) (C-20)	65	1:2000	Cell Signaling Tech.
Rabbit polyclonal anti-p38 MAPK	40	1:1000	Cell Signaling Tech.
Rabbit polyclonal anti-MAP Kinase (ERK-1, ERK-2)	42/44	1:10000	Cell Signaling Tech.
Rabbit polyclonal anti-mTOR	289	1:1000	Cell Signaling Tech.
Goat anti-mouse IgG/IgM-HRP conjugated			Sigma-Aldrich
Goat anti-rabbit IgG-HRP conjugated			Sigma-Aldrich

Table S2. List of primary and secondary antibodies used in WB.

Table S3.

Gene	Forward primer 5'-3'	Reverse primer 5'-3'
Fbxo32	GTCTTGGGAATGTACGACGC	GCGCTCCTTCGTACTTCCTT
Gapdh	GCCTTCCGTGTTCCTACCC	CAGTGGGCCCTCAGATGC
Myh2	AAACAGAAGCAGGAGCGACC	CACCGGCAGCCATTTGTAAG

Table S3. List of primers used in real-time PCR.

Table S4

	Change in myotube diameter, %		Change in myotube diameter, %		Change in myotube diameter, %
**TNFα/IFNγ**	**-41.3**	**Dex**	**-23.9**	**PBS**	**-46.4**
W. somnifera	+51.3	*P. ginseng.*	+20.8	*A. repens*	-13.4
P. ginseng	+16.3	*W. somnifera*	+20.3	*P. boldus*	-19.3
S. marianum	+7.8	*A. repens*	+5.9	*W. somnifera.*	-21.6
R. officinale	+6.1	*S. marianum*	+3.1	*T. foenum-graecum*	-22.5
P. boldus	-2.1	*S.chinensis*	-8.1	*P. ginseng*	-24.3
G. biloba	-6.4	*T. foenum-graecum*	-8.5	*U. dioica*	-37.4
S.chinensis	-6.8	*P. boldus*	-14.3	*S. marianum*	-46
U. dioica	-9.9	*G. biloba*	-17	*R. officinale*	-48
T. foenum-graecum	-15.6	*R. officinale*	-30	*S.chinensis*	-48.4
A.repens	-16.6	*U. dioica*	-32	*G. biloba*	-50.3

Table S4. C2C12 myotubes were treated with TNFα (tumor necrosis factor α)/IFNγ (interferon γ), Dex (dexamethasone) or PBS (phosphate buffered saline) in the absence or presence of the selected plant extracts for 48h (TNFα/IFNγ and Dex) or 16h (PBS) and IF for myosin heavy chain (MyHC)-II was performed. Reported are the percentages of myotube diameter changes in comparison to untreated myotube.

Supplementary Figure 1. C2C12 myotubes were treated with one-hundred different plant extract (100 µg/mL) for 48h. May-Grünwald/Giemsa staining was performed and total myotube area was measured by *Image J* software. The graph reports the percent changes in myotube area relative to untreated control.

Supplementary Figure 2. (A,B) C2C12 myotubes were treated with T (tumor necrosis factor α, 20 ng/mL)/I (interferon γ, 100 U/mL) (T/I) in the absence or presence of each selected 96 plant extracts (100 µg/mL) for 48h. May-Grünwald/Giemsa staining was performed and total myotube areas were measured by *Image J* software (A). The graph reports the percent changes in myotube area with respect to untreated control. Dotted line indicates the arbitrary cut off to select the extracts

able to impair T/I-dependent myotube area reduction up to 10% (A). Reported are representative images of the 17 extracts selected for further investigation. Bars, 500 µm (B).

Supplementary Figure 3. C2C12 myotubes were untreated or treated with dexamethasone (Dex, 1 µM) for 48h (A) or starved with PBS (phosphate buffered saline) for 16h (B) in absence or presence of the selected 10 plant extracts. Immunofluorescence (IF) analysis for myosin heavy chain (MyHC)-II was performed and myotube diameters were measured by *Image J* software. Reported are representative images with average myotube diameters (µm)±SD (standard deviation). Bars, 100 µm.

Supplementary Figure 4. Full ^{1}H NMR (Nuclear Magnetic Resonance) spectra of the most active extracts, with highlighted extended regions on top of the figure; diagnosis signals of: 1=alanine, 2=quinic acid, 3=aspartate, 4=malic acid, 5=citric acid, 6=sucrose, 7=*β*-glucose, 8=*α*-glucose, 9=caffeic acid, 10=shikim acid, 11=trigonelline.

Supplementary Figure 5. (A) C2C12 myotubes were treated with TNFα (tumor necrosis factor α, 20 ng/mL)/IFNγ (interferon γ, 100 U/mL) (T/I) in the absence or presence of ten selected plant extracts (100 µg/mL) for 48h and the expression of myosin heavy chain (MyHC)-II expression were analyzed by WB (western blotting). Reported are representative images. Reported are the relative densities with respect to tubulin. Statistical analysis was conducted using t-test *p<0.05, **p<0.01 significantly different from untreated control. (B) C2C12 myotubes were treated with T/I in the absence or presence of the 20 different herbal formulations (100 µg/mL) for 48h. May-Grünwald/Giemsa staining was performed and total myotube area was measured by *Image J* software. The graph reports the percent changes in myotube area relative to untreated control. Five formulations were able to counteract the reduction of the myotube area induced by T/I. Bars, 500 µm.

Supplementary Figure 6. C2C12 myotubes were treated with TNFα (tumor necrosis factor α, 20 ng/mL)/IFNγ (interferon γ, 100 U/mL) (T/I) (A,B) or dexamethasone (Dex, 1 µM) (C,D) in the absence or presence of WST (100 µg/mL). WB (western blotting) analysis was performed to verify p-ERKs (phospho-extracellular signal-regulated kinases), ERKs, p-p65 (phospho-NF-κB p65, nuclear factor kappa-light-chain-enhancer of activated B cells p65) and p65 levels after 6h, whereas p-mTOR (mammalian target of rapamycin), mTOR and MyoD (myoblast determination protein 1) expression were analyzed after 48h (A,C). Tubulin was used as protein loading control (A,C). Levels of *Myh2 (Myosin heavy chain 2)* and *Fbxo32* (*muscle atrophy F-box protein*) were analyzed after 24h by real-time PCR. (E) Myotubes were treated with WST and analyzed after 48h by IF (immunofluorescence) for myosin heavy chain (MyHC)-II. Reported are representative images

(A,C,E). Reported are the relative densities with respect to tubulin or total form of phosphorylated protein (A,C). Results are means±SD (standard deviation) (A-E). Statistical analysis was conducted using t-test *$p<0.05$, **$p<0.01$ significantly different from untreated control; $^{\#}p<0.05$ and $^{\#\#}p<0.01$ significantly different from T/I (A,B,E) or Dex (C,D).

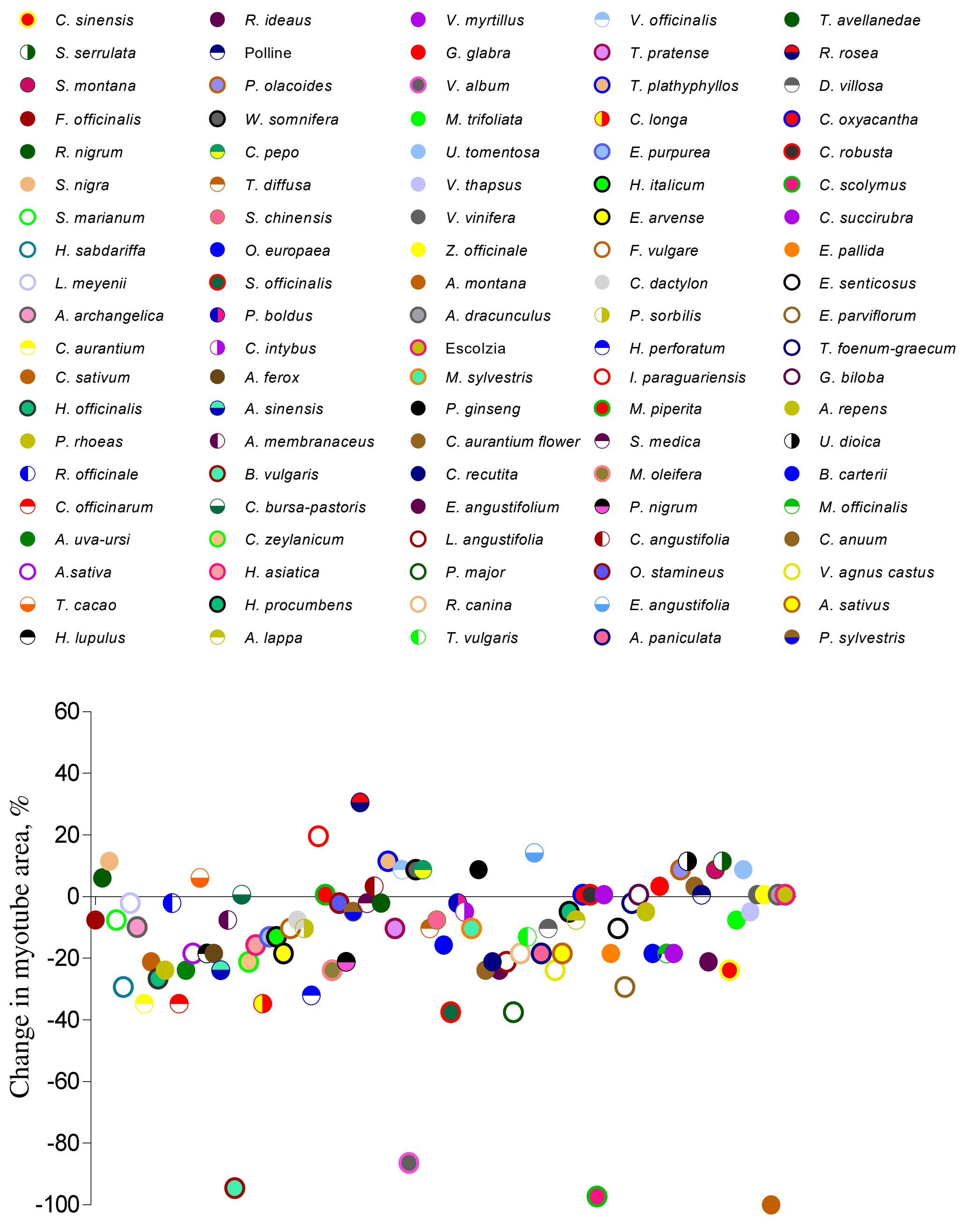

Supplementary Figure 1

A

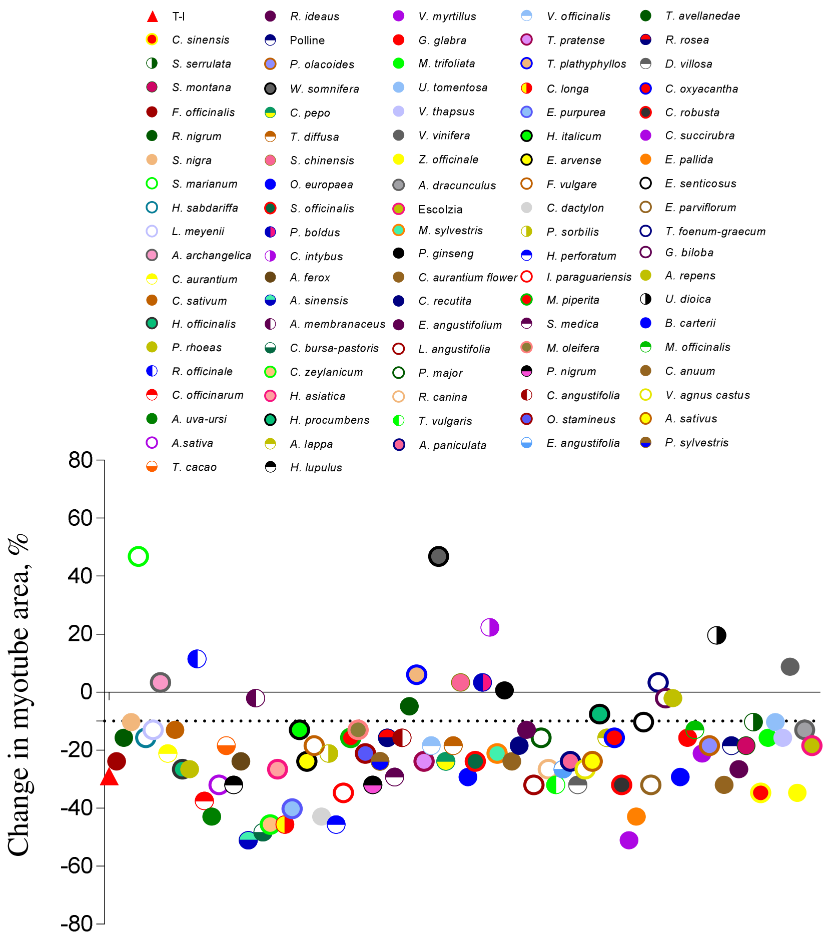

B

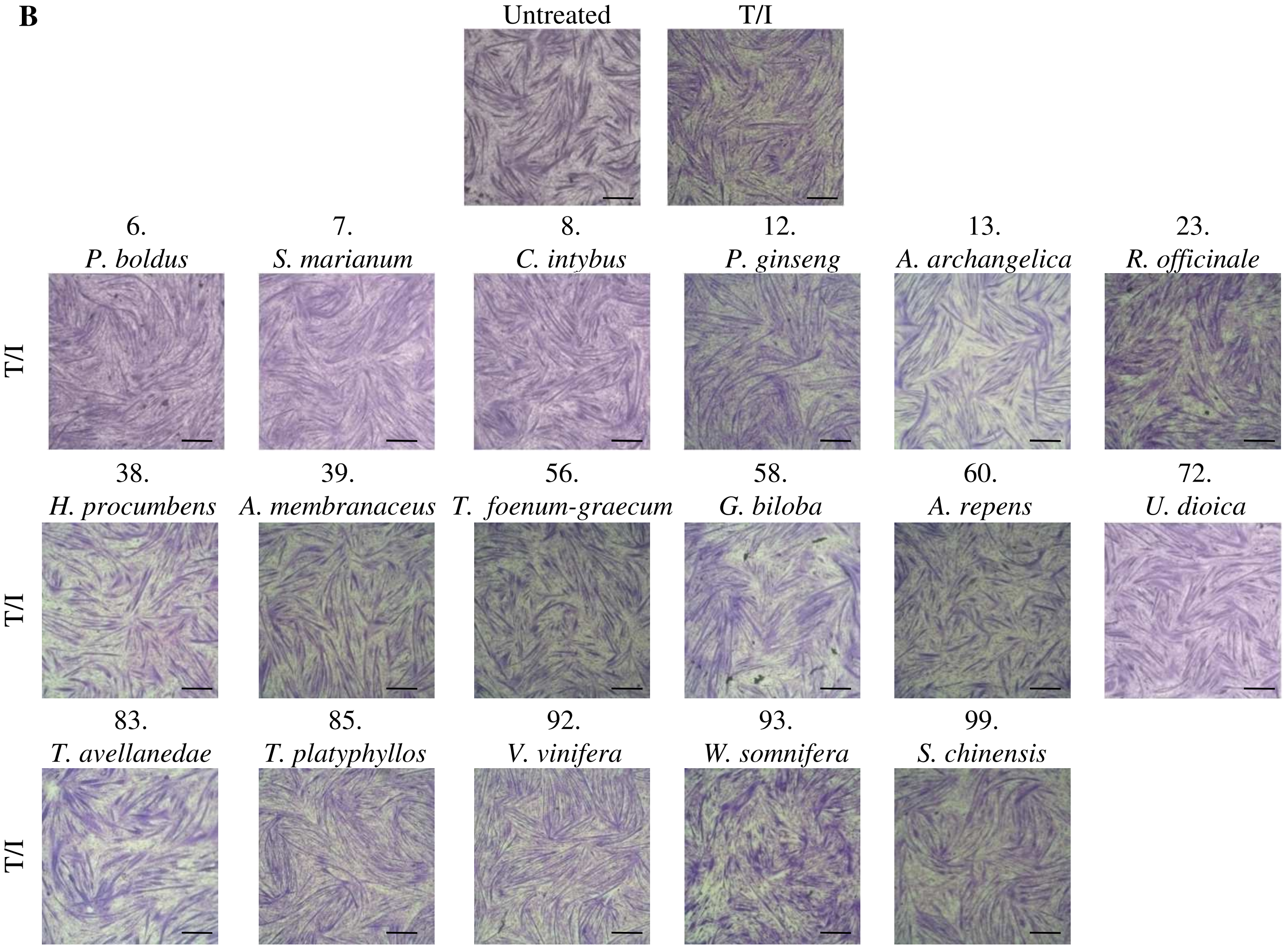

Supplementary Figure S2

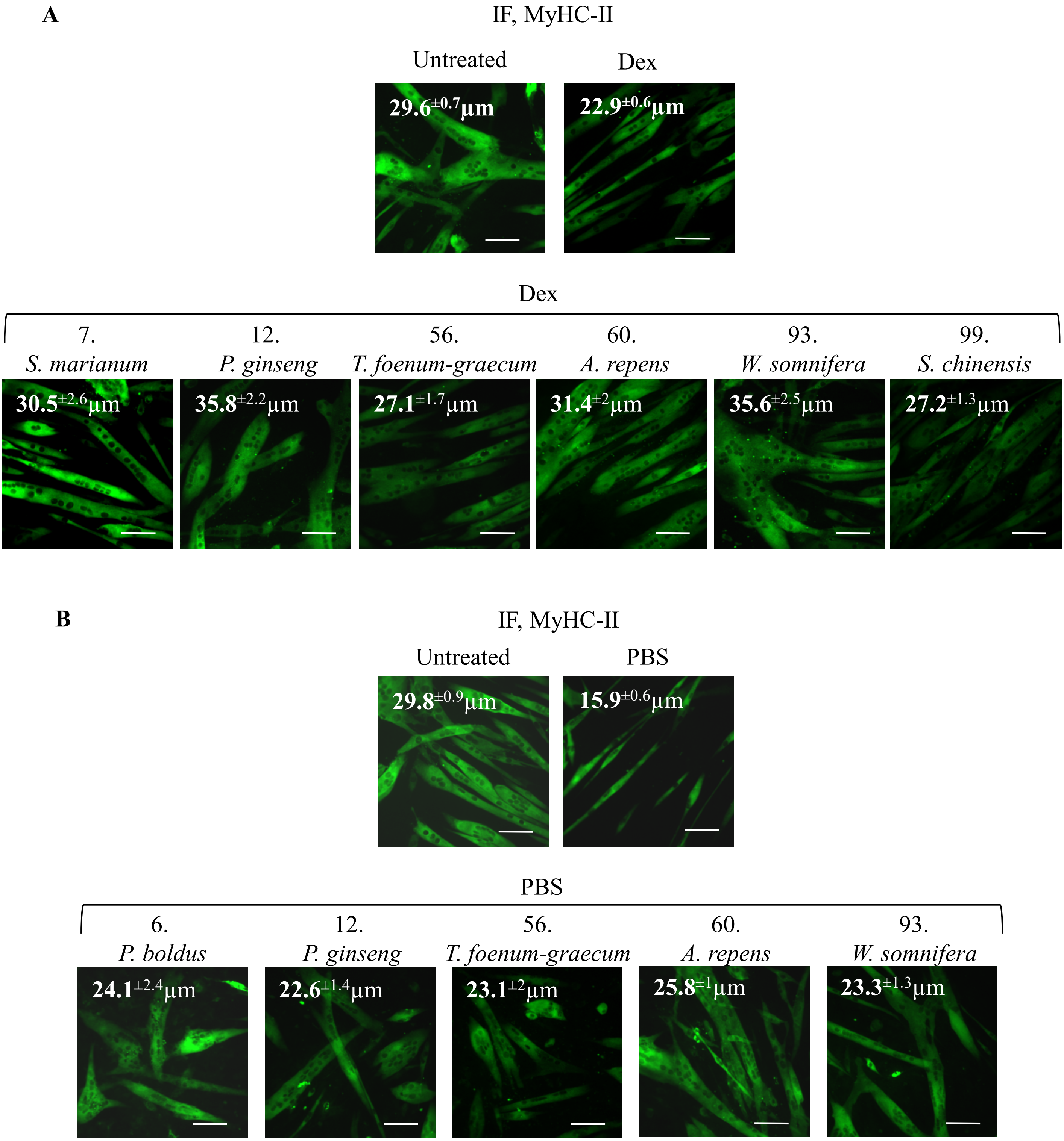

Supplementary Figure 3

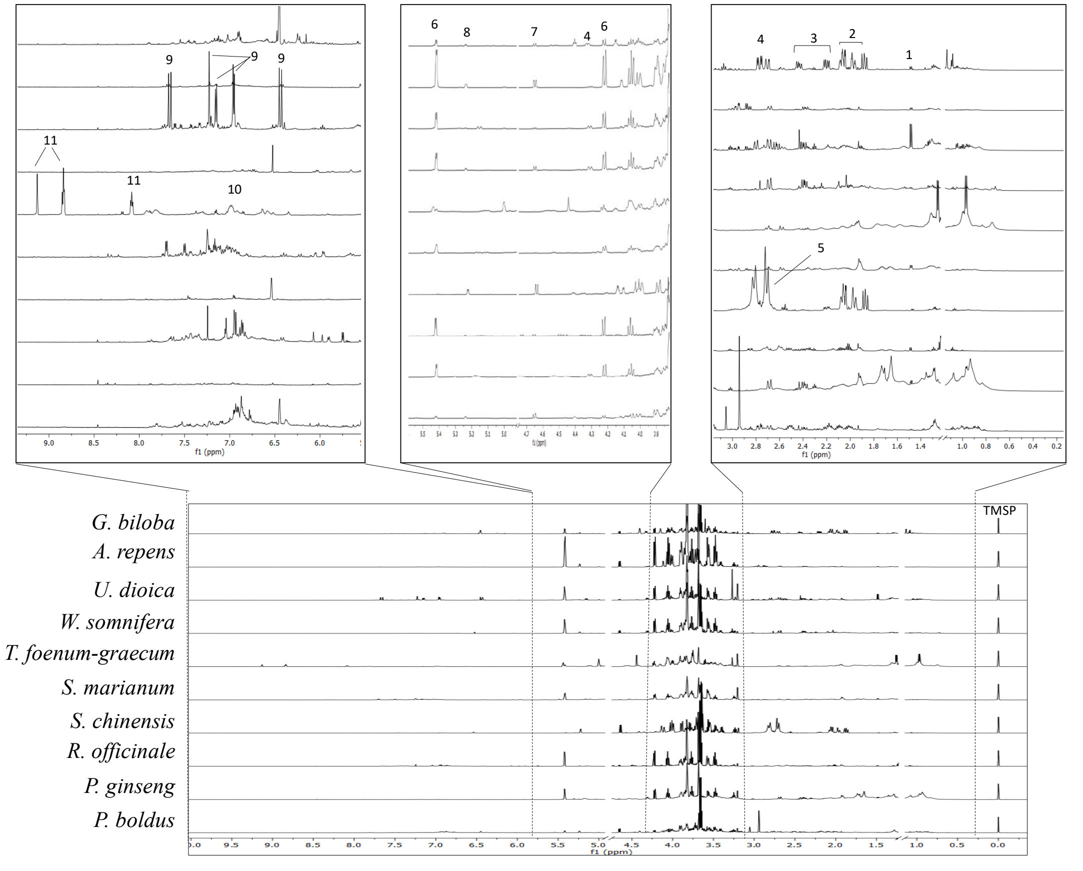

Supplementary Figure 4

A

6. *P. boldus*
7. *S. marianum*
12. *P. ginseng*
23. *R. officinale*
56. *T. foenum-graecum*
58. *G. biloba*
60. *A. repens*
72. *U. dioica*
93. *W. somnifera*
99. *S. chinensis*

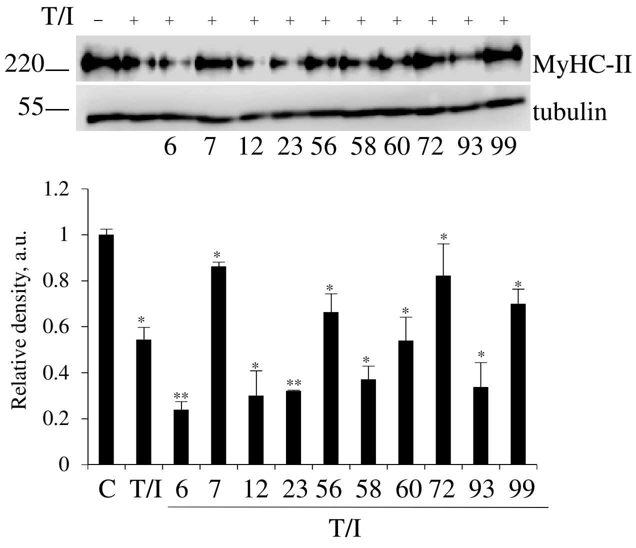

B

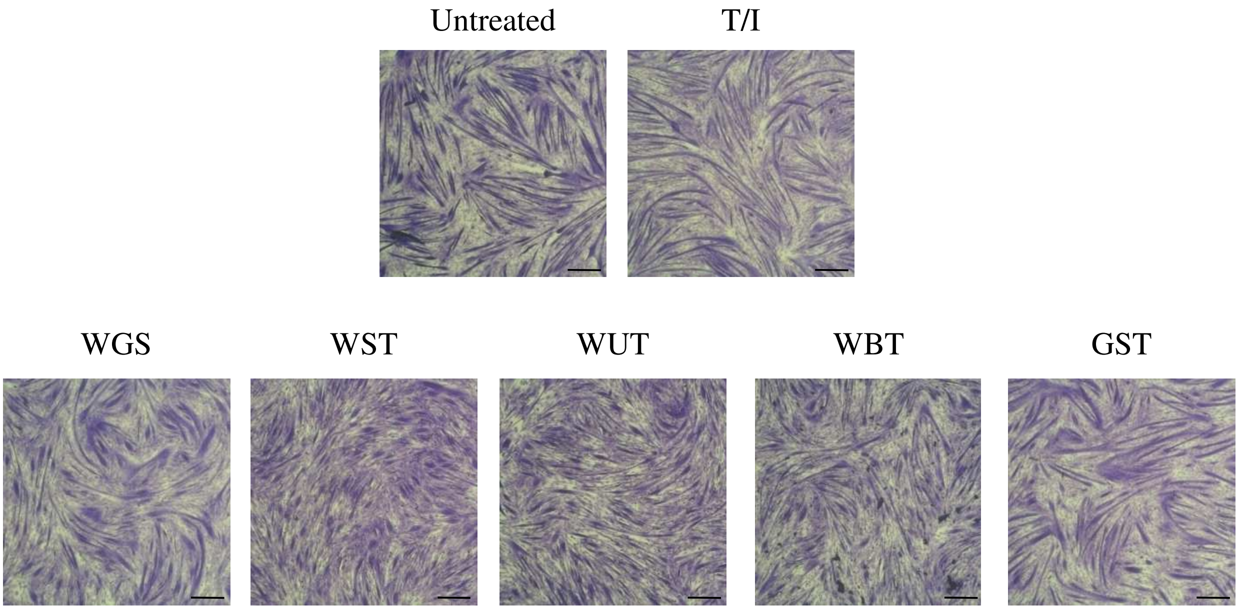

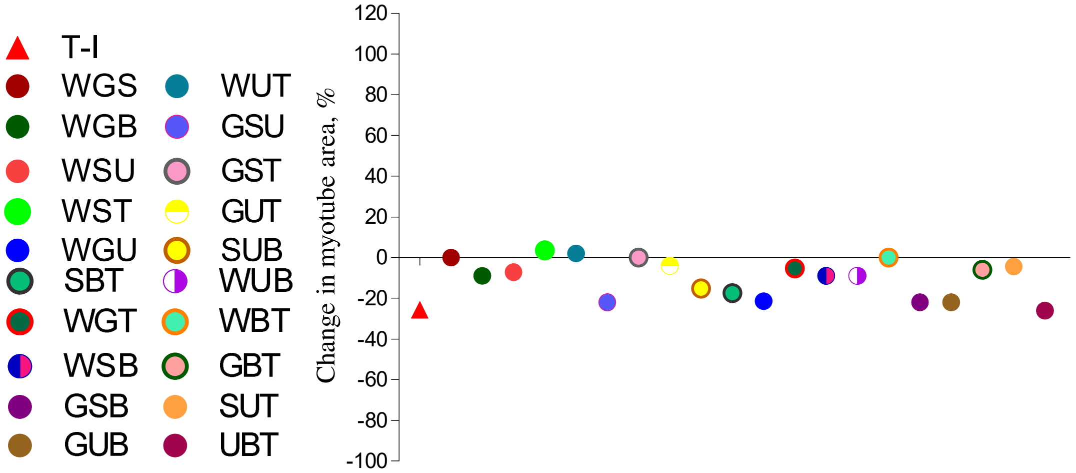

Supplementary Figure 5

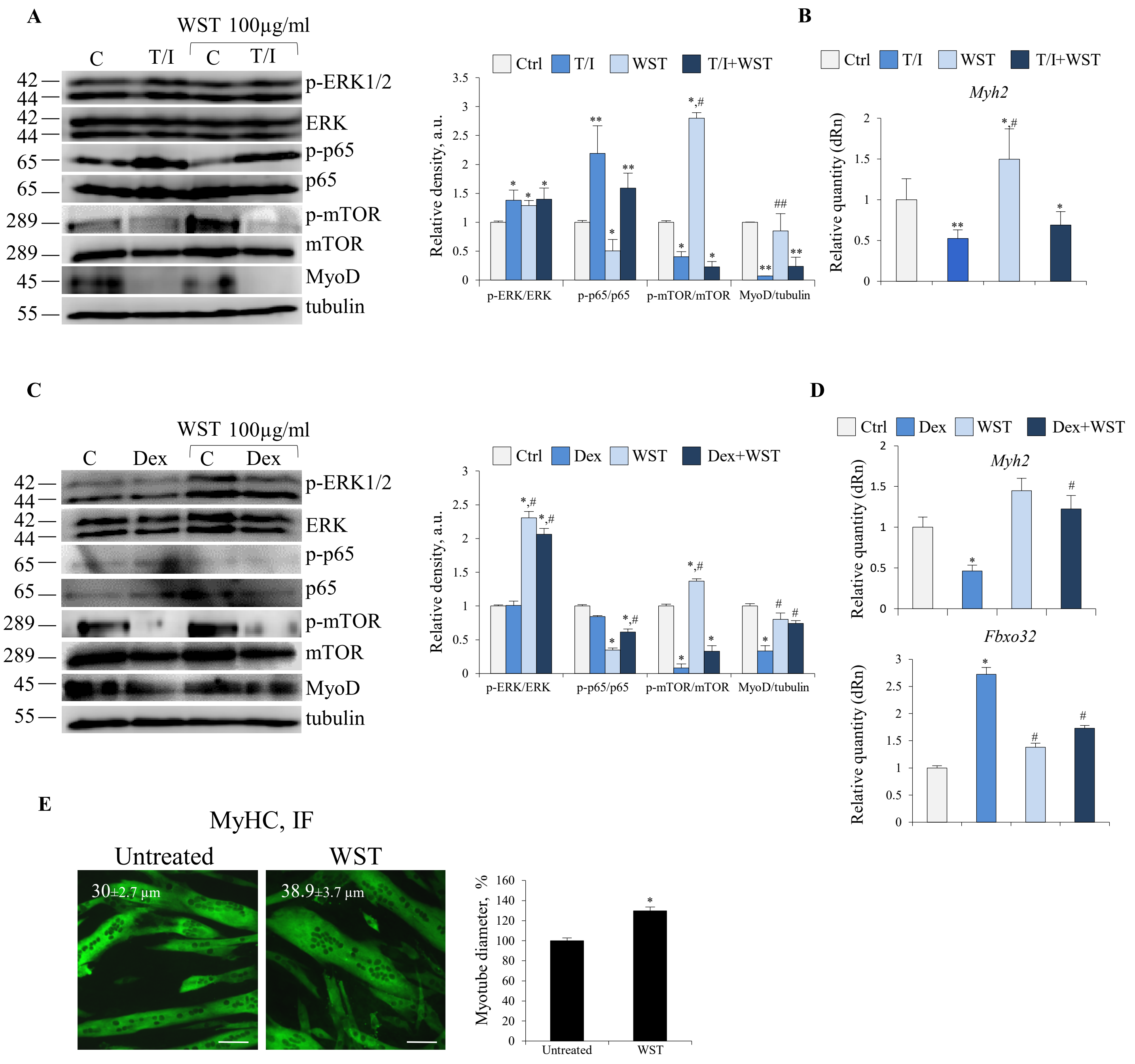

Supplementary Figure 6
